# *Cornus officinalis* Fruit Extract as an AMPK-Associated Mitochondrial Bioenergetic Modulator in Skin Aging Models

**DOI:** 10.3390/biomedicines14020403

**Published:** 2026-02-10

**Authors:** Rui Ye, Qianqian Wang, Le Du, Li Li, Fan Hu

**Affiliations:** 1 Department of Dermatology & Venerology, Center of Cosmetic Safety and Efficacy Evaluation and NMPA, Key Laboratory for Human Evaluation and Big Data of Cosmetics, West China Hospital Sichuan University, Chengdu 610041, China; rye@uniskin.com; 2 UNISKIN Research Institute on Skin Aging, Inertia Shanghai Biotechnology Co., Ltd., Shanghai 200021, China; ldu@uniskin.com; 3 DermaHealth Shanghai Biotechnology Co., Ltd., Shanghai 200021, China; 4 Department of Dermatology, Huashan Hospital, Fudan University, Shanghai 200031, China; wangqianqian@huashan.org.cn

**Keywords:** *Cornus officinalis*, AMPK, ATP, OXPHOS, fibroblast

## Abstract

**Background:** Mitochondrial dysfunction is a fundamental driver of skin aging, making the enhancement of cellular bioenergetics an important strategy in dermocosmetic innovation. *Cornus officinalis* fruit extract (COFE), standardized for iridoid glycosides, was investigated for its ability to modulate mitochondrial function and counteract photo-oxidative stress associated with skin aging. **Methods:** Human dermal fibroblasts were treated with COFE to evaluate mitochondrial bioactivation. Transcriptomic changes were assessed using RNA sequencing (RNA-seq), with key mitochondrial genes validated by qPCR. AMPK phosphorylation, intracellular ATP content, NAD^+^/NADH ratio, and mitochondrial membrane potential (ΔΨm) were quantified as functional indicators of mitochondrial performance. To examine anti-aging relevance, a reconstructed human epidermis model was challenged with UVA and retinol to induce photo-oxidative stress. COFE’s effects on inflammatory (IL-1α), hydration (AQP3), proliferation (Ki67), and barrier-related (PKCα) markers were subsequently analyzed. **Results:** COFE was associated with activation of AMPK signaling and coordinated upregulation of OXPHOS-related genes in dermal fibroblasts, increasing ATP by 30.00%, the NAD^+^/NADH ratio by 158.71%, and ΔΨm by 158.82%. It also reduced IL-1α and upregulated AQP3, Ki67, and PKCα in a UVA/retinol-challenged epidermis model. In vivo, a 1% COFE eye cream produced statistically significant improvements across hydration, barrier function, redness, skin tone, wrinkles, elasticity, and periorbital contour after 28 days. **Conclusions:** COFE functions as an AMPK-associated mitochondrial bioenergetic modulator that enhances cellular energy metabolism and mitigates photo-oxidative stress in skin-relevant experimental models. The concordance between mechanistic findings and clinical outcomes supports COFE as a promising anti-aging active ingredient for dermocosmetic applications.

## 1. Introduction

The skin is a metabolically active organ composed of distinct layers. The epidermis functions as a regenerative barrier against environmental insults, while the dermis provides structural support through extracellular matrix (ECM) synthesis [1,2,3]. These functions are maintained by highly active cells such as proliferating keratinocytes and fibroblasts responsible for extracellular matrix synthesis, both of which depend on mitochondrial oxidative phosphorylation (OXPHOS) as their primary source of adenosine triphosphate (ATP) [4,5,6]. ATP is produced through a series of respiratory chain complexes (I to V) located in the mitochondrial inner membrane, which coordinate electron transport and proton translocation to establish the proton gradient required for ATP synthesis [7,8].

Mitochondria also regulate redox balance, apoptosis, and cell signaling. However, intrinsic aging and chronic environmental exposure, such as ultraviolet (UV) radiation, can impair mitochondrial function [9,10,11,12,13]. This dysfunction results in reduced ATP synthesis, increased reactive oxygen species (ROS) production, loss of mitochondrial membrane potential (ΔΨm), and downregulation of genes involved in the respiratory chain [14,15,16]. These changes contribute to oxidative stress, cellular senescence, and ECM degradation, which are key features of photoaging.

Markers such as transcription factor A mitochondrial (TFAM) and peroxisome proliferator-activated receptor gamma coactivator 1alpha (PGC-1α) are commonly used as indicators of mitochondrial transcriptional regulation and maintenance, and are often found at reduced levels in aged or photoexposed skin [17,18,19,20]. While not direct intervention targets, these markers offer insight into mitochondrial health during cutaneous aging. AMP-activated protein kinase (AMPK) functions as a central energy sensor that helps maintain mitochondrial homeostasis. Its activation enhances OXPHOS gene expression and ATP production, especially under metabolic or oxidative stress [21,22,23]. As such, the AMPK-associated OXPHOS regulatory axis represents a promising target for restoring skin cellular bioenergetics during aging and environmental challenge.

Maintaining mitochondrial function in both the epidermis and dermis is essential for skin vitality. Strategies that improve OXPHOS activity, restore redox homeostasis, and preserve energy metabolism have shown promise for anti-aging skincare applications [24,25]. Phytochemicals from traditional medicinal plants are increasingly explored as bioactive compounds that protect mitochondrial integrity.

*Cornus officinalis Sieb. et Zucc*. (Asiatic dogwood), widely used in traditional Chinese medicine, contains iridoid glycosides (such as morroniside and loganin), flavonoids, and phenolic acids. These compounds have been associated with antioxidant, anti-inflammatory, and mitochondrial-supporting effects. Extracts from *C. officinalis* have shown potential in reducing oxidative damage and preserving mitochondrial functional performance in various biological models [26,27,28,29,30]. *Cornus officinalis* has been extensively studied due to its well-established biological activities. The Qinling region in Shaanxi Province, a major production area of *C. officinalis*, presents a distinctive ecological environment that justifies further investigation into the potential skin-related bioactivities of its fruits. Nevertheless, the mechanisms by which *C. officinalis* influences mitochondrial function in the skin remain insufficiently characterized.

In this study, we evaluated the mitochondrial bioenergetic–modulating effects of a standardized *Cornus officinalis* fruit extract (COFE) sourced from the Qinling Mountains. Through transcriptomic profiling, network pharmacology, and functional assays in human skin cells, we examined whether COFE could modulate mitochondrial bioenergetic function, improve ATP-associated readouts, and protect against UV-induced mitochondrial dysfunction. These results provide mechanistic insights into the anti-aging potential of COFE and its relevance to skin bioenergetic health. To explore these mitochondrial regulatory effects, we established an integrated workflow combining network pharmacology, transcriptomic analysis, and functional validation in dermal fibroblasts, and further evaluated their exploratory translational relevance in vivo using a 1% COFE eye cream, as the periocular region represents a physiologically vulnerable site characterized by thin epidermal architecture, limited lipid content, and heightened susceptibility to oxidative and photo-induced aging [31,32].

## 2. Methods

### 2.1. Cornus officinalis Fruit Extract Preparation

*Cornus officinalis* fruits collected from Qinling Mountains (Xi’an, China) were dried, ground into powder, and extracted with 60% ethanol under enzymatic assistance using a composite enzyme system composed of pectinase and cellulase, which facilitates degradation of plant cell wall polysaccharides and enhances the release of intracellular iridoid glycosides. After enzymatic inactivation, the extract was concentrated and further purified using a polyamide resin column. The final standardized extract was designated as COFE (*Cornus officinalis* fruit extract). All experimental assays in this study were performed using the same batch of COFE to ensure consistency.

### 2.2. Phytochemical Profiling of COFE

The chemical constituents of *Cornus officinalis* fruit extract (COFE) were analyzed using LC-MS on an ACQUITY UPLC I-Class system coupled with a Q Exactive mass spectrometer. Raw data were processed with Progenesis QI v3.0 for peak detection, alignment, and normalization. Compound identification was conducted based on accurate mass, MS/MS fragmentation patterns, and isotopic distribution, using a curated Traditional Chinese Medicine (TCM) compound database containing over 5000 known phytochemicals. Representative chemical classes identified included iridoids, flavonoids, phenolic acids, and terpenoids.

### 2.3. Functional Annotation and Network Pharmacology Analysis

Putative targets of the identified compounds were predicted using publicly available chemoinformatics databases. The predicted compound–target associations were used to construct a compound–target–pathway (C–T–P) network. Gene Ontology (GO) enrichment analysis was subsequently performed on the predicted targets to identify associated biological processes. Enriched GO terms were recorded and used to inform downstream experimental validation.

### 2.4. Cell Viability Assay

Human dermal fibroblasts (Batch No. 230131, Guangdong Boxi Biotechnology Co., Ltd., Dongguan, China) were seeded into 96-well plates at approximately 60% confluence and incubated at 37 °C in a humidified atmosphere with 5% CO_2_ for 24 h. Cells were then treated with various concentrations of *Cornus officinalis* fruit extract (COFE) samples (0.078–10.00%, *v*/*v*) dissolved directly in phosphate-buffered saline (PBS) for 24 h. After treatment, MTT solution (M56655, 0.5 mg/mL, Sigma, St. Louis, MO, USA) was added to each well and incubated for 4 h. Subsequently, DMSO (D4540-1L, Sigma, St. Louis, MO, USA) was added to dissolve the formazan crystals. Cell viability was determined by measuring the absorbance at 490 nm using a microplate reader (Epoch, BioTek, Winooski, VT, USA). In parallel, morphological changes were observed using phase-contrast microscopy (CKX53, Olympus, Tokyo, Japan) to qualitatively assess cell health, including alterations in cell shape, adherence, and density. This provided supportive visual evidence for the MTT-based viability assessment.

### 2.5. RNA Isolation, cDNA Library Preparation, and Sequencing

Cells were divided into three groups: blank control (BC), retinol-treated, and COFE-treated. After 24 h treatment, cells were collected for transcriptome analysis. Total RNA was extracted using TRIzol reagent (Invitrogen, Carlsbad, CA, USA). RNA concentration and purity were assessed using a NanoDrop spectrophotometer (Thermo Fisher Scientific, Waltham, MA, USA) and Qubit Fluorometer (Thermo Fisher Scientific, Waltham, MA, USA), and RNA integrity was evaluated using an Agilent 2100 BioAnalyzer (Agilent Technologies, Santa Clara, CA, USA). Only samples with an RNA Integrity Number (RIN) ≥ 7.0 and a 28S:18S rRNA ratio ≥ 1.8 were used for library preparation.

For each sample, 1 μg of total RNA was subjected to mRNA enrichment using the NEBNext^®^ Poly(A) mRNA Magnetic Isolation Module (NEB) to (New England Biolabs, Ipswich, MA, USA) selectively capture polyadenylated transcripts. Sequencing libraries were constructed using the NEBNext^®^ Ultra™ RNA Library Prep Kit for Illumina (NEB) according to the manufacturer’s instructions (New England Biolabs, Ipswich, MA, USA). Briefly, enriched mRNA was fragmented to approximately 200–300 bp, followed by first-strand cDNA synthesis using random primers and second-strand cDNA synthesis. cDNA fragments were end-repaired, A-tailed, ligated to Illumina sequencing adapters, PCR-amplified, purified, and enriched to generate final libraries.

Library quality and size distribution were validated using the Agilent 2100 BioAnalyzer (Agilent Technologies, Santa Clara, CA, USA), and libraries were quantified using the KAPA Library Quantification Kit (KAPA Biosystems, Wilmington, MA, USA). Sequencing was performed on an Illumina NovaSeq (Illumina, San Diego, CA, USA) platform to generate 150 bp paired-end reads. Raw sequencing data quality was evaluated using FastQC (v0.11.2). Adapter sequences, poly(A) tails, and low-quality bases were removed using Cutadapt (v1.18). Reads containing more than 5% ambiguous nucleotides (N) or with fewer than 70% of bases having a Phred quality score ≥ 20 were discarded. High-quality paired-end reads were aligned to the reference genome using HISAT2 (v2.2.0). Differentially expressed genes (DEGs) were identified using a threshold of |log_2_ fold change| ≥ 1 and adjusted *p*-value ≤ 0.05. Functional enrichment analyses were subsequently performed to identify pathways related to mitochondrial bioenergetics, oxidative phosphorylation, redox regulation, and extracellular matrix remodeling.

### 2.6. ATP Synthesis Assay

Cells were seeded into 24-well plates at approximately 60% confluence and incubated overnight at 37 °C with 5% CO_2_. Treatments were applied with three replicates per group: blank control (BC), positive control (quercetin, 300 nM; Q111274-50g, Aladdin, Shanghai, China), and COFE at 0.5% and 1.0%. Quercetin was initially solubilized in DMSO and subsequently diluted in PBS to the working concentration, resulting in a final DMSO concentration of 0.1% (*v*/*v*). After 24 h of incubation, cells were washed twice with 2 mL of PBS. ATP levels were measured using the Cell Meter ATP Live Cell Assay Kit (BX43, Baiying, Xi’an, China). This assay is based on an ATP-responsive fluorescent dye system that selectively detects intracellular ATP content in live cells, and does not rely on mitochondrial membrane potential–dependent probes. Cellular fluorescence was observed under a fluorescence microscope (BX43, Olympus, Tokyo, Japan). The relative percentage change was calculated using the following formula: $Percentage\;change(\%)\;=\;\frac{{IOD}_{COFE}\;-\;{IOD}_{BC}}{{IOD}_{BC}}\times 100\%$, where IOD stands for integrated optical density, defined as the sum of pixel intensities within the region of interest. The IOD value was normalized to the BC group, which was set to 1.0.

### 2.7. In Vitro Gene Expression Assessment

Cells cultured in 6-well plates were harvested for quantitative reverse transcription polymerase chain reaction (qRT-PCR) analysis. Total RNA was extracted using TRIzon reagent (CW0580S, Cwbio, Beijing, China), quantified with a Qubit Flex Fluorometer (Invitrogen, Carlsbad, CA, USA), and reverse-transcribed into cDNA using the HiFiScript cDNA Synthesis Kit (CW2569M, Cwbio, Beijing, China). Real-time PCR was performed using the UltraSYBR Mixture (CW0957M, Cwbio, Beijing, China) on a qTOWER384 real-time PCR system (Analytik Jena AG, Jena, Germany). All reactions were conducted in duplicate. Primer sequences (synthesized by Tsingke Biotech, Beijing, China) are listed in Table 1. Relative gene expression levels were calculated using the 2^−ΔΔCt^ method.

### 2.8. Western Blot Analysis of AMPK Signaling Protein

HDFs were harvested and then lysed with RIPA lysis buffer (P0013B, Beyotime, Shanghai, China) containing PMSF (1:100, P0100 Solarbio, Beijing, China) and phosphatase inhibitors (1:50, P1082, Beyotime, Shanghai, China). The protein concentration was assayed using a BCA protein assay kit (BL521A, Biosharp, Beijing, China). Proteins were electrophoretically separated on SDS-PAGE gels (10% for β-actin, AMPK and phospho-AMPK) and then transferred onto PVDF membranes (IPVH00010, Millipore, Burlington, MA, USA). After blocking with 5% bovine serum albumin (BSA) at room temperature (RT) for 2 h, the membranes were incubated with primary antibodies overnight at 4 °C. The primary antibodies included anti-β-actin (ab8227, 1:2500, Abcam, Cambridge, UK), anti-AMPK (2532, 1:1000, Cell Signaling, Danvers, MA, USA), anti-phosphoAMPK (2535, 1:1000, Cell Signaling, Danvers, MA, USA). After being washed in TBS-T (0.05% Tween-20), the membranes were incubated with the species-appropriate HRP-conjugated secondary antibody at RT for 1 h. The second antibody was goat-anti-rabbit IgG (H + L) (31460, 1:10,000; Thermo, Waltham, MA, USA). Membranes were subsequently washed in TBS-T and immunodetected with ECL reagents (P0010S, Beyotime, Shanghai, China). All the bands were analyzed by Fiji ImageJ software (Rawak Software Inc., Stuttgart, Germany). As a canonical AMPK agonist widely used in metabolic and mitochondrial research, metformin serves as a mechanistic validation control for AMPK–OXPHOS pathway activation.

### 2.9. NAD^+^/NADH Assay

When cell confluence in 6-well plates reached approximately 50%, treatments were initiated. Each well was supplemented with 2 mL of medium: standard culture medium for the blank and negative control groups, medium containing quercetin (300 nM) for the positive control group, and medium containing COFE at 0.5% or 1.0% (*v*/*v*) for the sample groups. Cells were then incubated at 37 °C in a humidified atmosphere with 5% CO_2_ for 24 h. For UVA exposure (Philips, PL-S 9W/10/2P, Amsterdam, Netherlands), all groups except the blank control were irradiated at a dose of 30 J/cm^2^ (15.22 mW/cm^2^). After irradiation, cells were returned to the incubator and cultured for an additional 24 h. Cells were subsequently harvested, and intracellular NAD^+^ and NADH levels were quantified using a commercial NAD^+^/NADH Assay Kit (S0175, Beyotime, Shanghai, China), and expressed as protein-normalized concentrations rather than absolute intracellular values.

### 2.10. Mitochondrial Membrane Potential (ΔΨm) Assay

Cells were treated and UVA-irradiated as described in the NAD^+^/NADH assay. After 24 h of post-irradiation incubation, mitochondrial membrane potential was assessed using Image-iT™ TMRM reagent (I34361, Invitrogen, Carlsbad, CA, USA) following the manufacturer’s protocol. This assay is mechanistically independent of intracellular ATP content measurement. Briefly, cells were incubated with the TMRM working solution at 37 °C, then gently washed with PBS. Fluorescence signals were captured using a fluorescence microscope (BX43, Olympus, Tokyo, Japan), and ΔΨm changes were evaluated based on fluorescence intensity. Quantification was performed using Image-Pro Plus 6.0 (IPP). The percentage change in fluorescence was calculated using the following formula: $Percentage\;change\left(\%\right)\;=\;\frac{{IOD}_{UVA+COFE}\;-\;{IOD}_{UVA}}{{IOD}_{UVA}}\;\times \;100\%$. The IOD value was normalized to the BC group, which was set to 1.0. Percentage values are therefore intended to represent relative recovery magnitude rather than absolute intracellular levels.

### 2.11. ROS Detection in UVA-Irradiated Fibroblasts Treated with COFE

Fibroblasts were seeded in 6-well plates and pretreated with COFE (0.5% or 1%, *v*/*v*) or vitamin E (0.1%, positive control) for 30 min. All groups except the blank control (BC) were then exposed to UVA irradiation at 30 J/cm^2^ (15.22 mW/cm^2^). Following UVA exposure, cells were incubated with 10 μM DCFH-DA (S0033M, Beyotime, Shanghai, China) for 30 min at 37 °C, then fixed with 4% paraformaldehyde (BL539A, Biosharp, Beijing, China). Intracellular ROS levels were visualized by fluorescence microscopy (DM2500, Leica, Wetzlar, Germany) and quantified using integrated optical density (IOD) via Image-Pro Plus 6.0. Each condition was tested in triplicate. ROS scavenging efficiency was calculated as follows: $Percentage\;change(\%)\;=\;\frac{{IOD}_{UVA}\;-\;{IOD}_{UVA+COFE}}{{IOD}_{UVA}}\;\times \;100\%$. The IOD value was normalized to the BC group, which was set to 1.0. Percentage values are therefore intended to represent relative recovery magnitude rather than absolute intracellular levels.

### 2.12. UVA/Retinol-Induced Skin Stress Model and COFE Intervention

The EpiKutis^®^ 3D epidermal model was used to evaluate the protective effects of COFE against UVA- and retinol-induced skin stress. A 0.1% retinol solution (12.5 μL, R7632, Sigma, St. Louis, MO, USA) was topically applied to the tissue surface in the NC, PC, and COFE groups for 30 min, followed by UVA irradiation (10 J/cm^2^, 19.25 mW/cm^2^). Retinol was initially solubilized in ethanol and diluted in PBS to the working concentration, with a final ethanol concentration of 1% (*v*/*v*). Immediately after irradiation, 12.5 μL of the respective test solution—vehicle (NC), Vitamin E (PC), or COFE—was evenly applied. Tissues were treated once daily for three consecutive days. After the final treatment, they were incubated for an additional 24 h. Culture supernatants were collected for IL-1α quantification via ELISA (ab100560, Abcam, Cambridge, UK). Tissues were then fixed in 4% paraformaldehyde at 4 °C for 24 h and processed for immunohistochemistry using antibodies against AQP3 (ab125219, Abcam, Cambridge, UK), Ki67 (GT209407, GeneTech, South San Francisco, CA, USA), and PKCα (ab32376, Abcam, Cambridge, UK). Secondary detection was performed using goat anti-mouse IgG H&L (Alexa Fluor^®^ 488, ab150117, Abcam, Cambridge, UK), and fluorescence images were acquired for marker expression analysis. Experimental groups included blank control (BC), negative control (Retinol + UVA, NC), positive control (Retinol + UVA + PC), and the COFE group (Retinol + UVA + COFE).

### 2.13. Statistical Analysis

Data are expressed as mean ± SD, except qPCR results, which are shown as mean ± SE. Statistical analysis was performed using GraphPad Prism 8.0. One-way ANOVA with Tukey’s post hoc test was used for multiple comparisons. For two-group comparisons, unpaired two-tailed Student’s *t*-test was applied. *p* < 0.05 was considered statistically significant. IOD values and Western blot bands were quantified using Image-Pro Plus 6.0. Relative gene expression was analyzed using the 2^−ΔΔCt^ method.

### 2.14. Clinical Assessment of an Eye Cream Formulated with Cornus officinalis Fruit Extract

A single-arm before–after clinical study was conducted at Beijing Ewish Testing Technology Co., Ltd. (Beijing, China). Thirty-four healthy Chinese volunteers aged 18–40 years (mean age: 31.68 ± 1.21 years) with visible periorbital dullness, laxity, and wrinkles were enrolled. All participants had a Baumann Skin Type Questionnaire score ≥30, indicating sensitive skin. Exclusion criteria included pregnancy or lactation, plans for pregnancy, systemic disease, severe dermatological conditions, known allergies, or recent medical or cosmetic treatments.

The study protocol was approved by the Ewish Ethics Committee for Clinical Research (Project No. KY202506115). All participants provided written informed consent prior to enrollment. The test product was a leave-on water-in-silicone eye cream formulated for periocular application. COFE was incorporated at 1% (*w*/*w*) as the primary bioactive ingredient, while other formulation components consisted of conventional cosmetic excipients, including humectants and silicone-based ingredients, used for emulsion stability, moisturization, and skin compatibility. No additional actives targeting mitochondrial or anti-aging pathways were intentionally included.

Participants applied the eye cream to the periorbital area twice daily for 28 consecutive days. Instrumental evaluations were performed at baseline (D0), Day 7 (D7), and Day 28 (D28). Before each assessment, participants washed their face and acclimated for ≥30 min in a controlled environment (21 ± 1 °C; 50 ± 10% relative humidity).

Stratum corneum hydration was measured using a Corneometer CM825; transepidermal water loss (TEWL) using a Tewameter TM300; skin elasticity (R2) and firmness (F4) using a Cutometer MPA580; lower-eyelid glossiness using a Glossymeter GL200; hemoglobin index using a Mexameter MX18; and skin roughness (SEr) of the lateral canthus using a VisioScan VC20 Plus (all Courage & Khazaka, Cologne, Germany). Standardized facial images were acquired using VISIA^®^ 6 (Canfield, Parsippany, NJ, USA) and analyzed with Image-Pro^®^ Plus (v7.0.1) to quantify lower-eyelid L* value, skin tone evenness, and red-area proportion. Three-dimensional wrinkle number and volume (crow’s feet and under-eye wrinkles) were assessed using AEVA-HE (EO-TECH, Marcoussis, France).

Paired-sample *t*-tests were applied to normally distributed variables, and Wilcoxon signed-rank tests to non-normal data. Statistical significance was set at *p* < 0.05.

## 3. Results

### 3.1. Phytochemical Composition of COFE Suggests Mitochondrial and ECM-Targeted Potential

**Figure 1:**
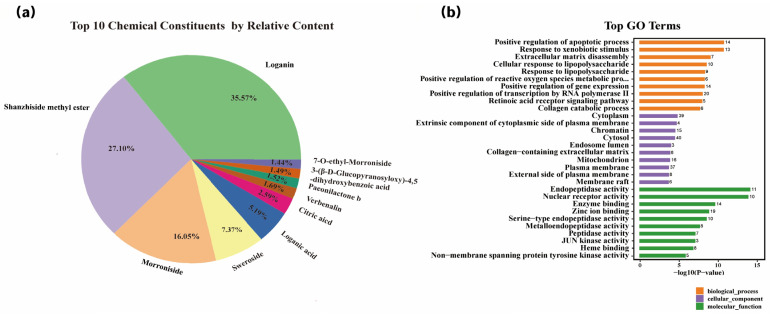
Phytochemical composition and GO enrichment analysis of COFE. (**a**) Top 10 chemical constituents of *Cornus officinalis* fruit extract (COFE), identified by Progenesis QI v3.0 based on normalized peak area. Iridoid glycosides such as loganin, shanzhiside methyl ester, and morroniside were among the most abundant compounds. (**b**) Gene Ontology (GO) enrichment analysis of overlapping targets between COFE-derived phytochemicals and skin-associated genes. Enriched GO terms are grouped into biological process (BP, orange), cellular component (CC, purple), and molecular function (MF, green), with statistical significance represented as −log_10_(*p*-values).

Phytochemical profiling (Figure 1a) identified key constituents in *Cornus officinalis* fruit extract (COFE), including loganin, shanzhiside methyl ester, and morroniside, which ranked among the top peaks based on relative abundance. These iridoid glycosides, along with other identified compounds such as loganic acid, sweroside, paeonolactone B, and verbascoside, have been implicated in antioxidant, anti-inflammatory, and mitochondrial-supportive activities in prior studies.

GO enrichment of their predicted targets (Figure 1b) revealed pathways related to oxidative stress response, mitochondrial regulation, and extracellular matrix (ECM) remodeling. While derived from in silico prediction, these data provided rationale for downstream experimental validation. Notably, many enriched terms-particularly those linked to oxidative phosphorylation and redox homeostasis-aligned with AMPK-related mechanisms later confirmed via transcriptomic and functional studies.

### 3.2. COFE Activates AMPK and Enhances Mitochondrial Bioenergetics Through Multi-Gene Regulation

To determine the optimal dosage range for mechanistic studies, cytocompatibility of COFE was first evaluated. MTT assay results showed that fibroblast viability remained at 90.06% under 1.25% COFE treatment, exceeding the commonly accepted 90% safety threshold. Both cell viability assays and microscopic evaluation confirmed the absence of morphological abnormalities at this concentration, supporting the cytocompatibility of COFE up to 1.25%. Based on these findings, concentrations of 0.5% and 1% were selected for subsequent functional assays.

**Figure 2:**
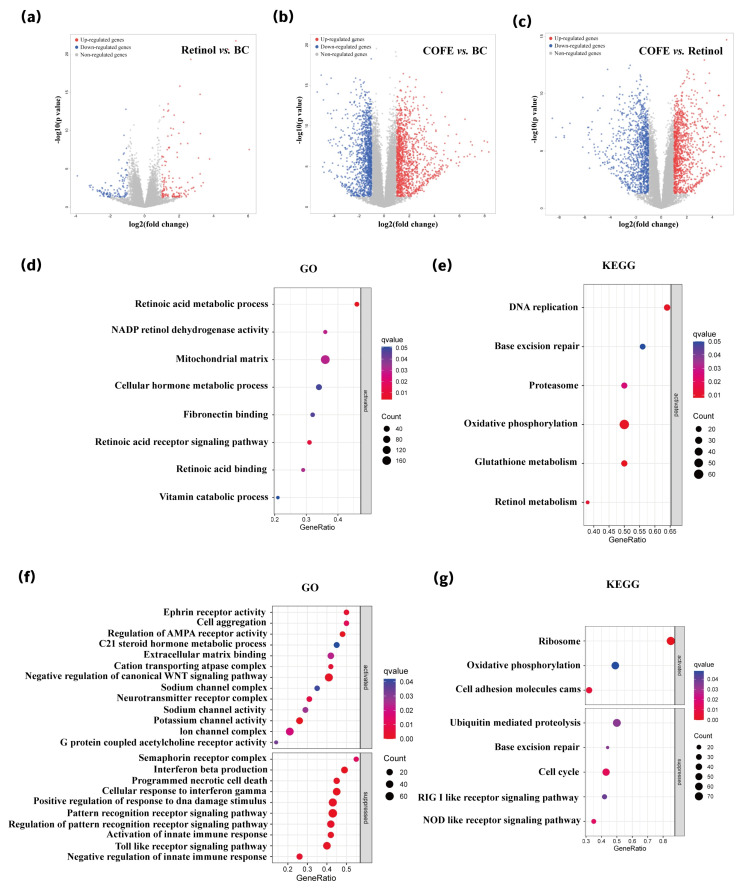
Transcriptomic profiling and enrichment analysis of COFE- and retinol-treated fibroblasts. (**a**–**c**) Volcano plots of differentially expressed genes (DEGs): (**a**) Retinol vs. blank control (BC), (**b**) COFE vs. BC, (**c**) COFE vs. Retinol. Red and blue dots represent significantly upregulated and downregulated genes, respectively (|log_2_FC| > 1, adjusted *p*-value < 0.05). (**d**,**e**) GO (**d**) and KEGG (**e**) enrichment analyses of upregulated genes in the retinol group compared to BC. (**f**,**g**) GO (**f**) and KEGG (**g**) enrichment analyses of upregulated genes in the COFE group compared to BC. Dot size represents the number of enriched genes; color indicates q-value; GeneRatio refers to the proportion of mapped genes relative to total DEGs.

To determine whether COFE modulates mitochondrial function, transcriptomic profiling and mechanistic validation were performed. RNA-seq results demonstrated broad transcriptional activation, particularly in pathways related to ECM organization, hormone metabolism, and immune modulation (Figure 2f). KEGG analysis further confirmed enrichment in oxidative phosphorylation, base excision repair, and cell cycle regulation (Figure 2g). At the gene level, COFE upregulated several key components of the oxidative phosphorylation pathway, including *COX6B2*, *ATP6V0B*, and *NDUFA4L2*, as well as multiple ECM-associated genes.

**Figure 3:**
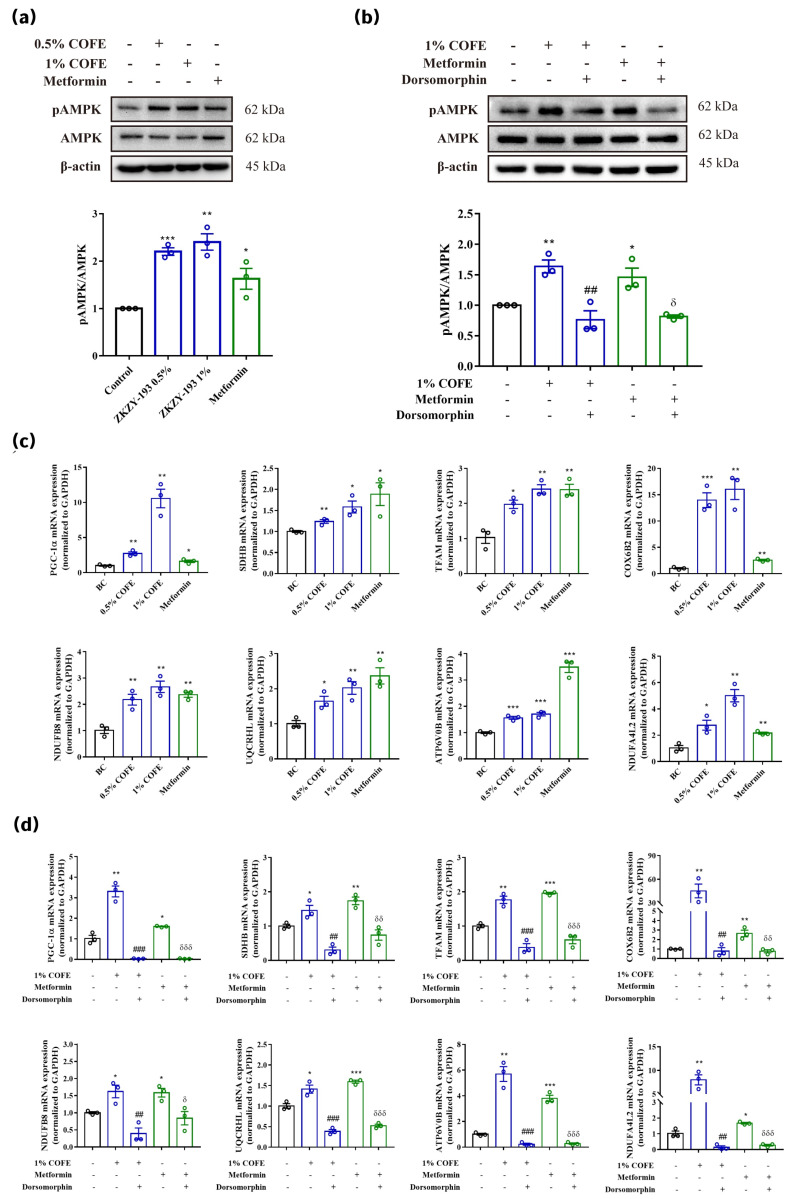
COFE activates AMPK signaling and upregulates mitochondrial-related gene expression in human dermal fibroblasts. (**a**) Western blot analysis showing the expression levels of phosphorylated AMPK (pAMPK) and total AMPK in fibroblasts treated with 0.5% or 1% COFE, or 1 mM metformin. The bar graph below depicts the pAMPK/AMPK ratio. (**b**) Western blot analysis of pAMPK and AMPK protein levels following treatment with 1% COFE, metformin, or COFE + dorsomorphin (AMPK inhibitor). (**c**) RT-qPCR analysis of mitochondrial biogenesis and oxidative phosphorylation-related genes (PGC-1α, SDHB, TFAM, COX6B2, NDUFB8, UQCRHL, ATP6V0B, and NDUFA4L2) following treatment with COFE or metformin. (**d**) RT-qPCR analysis of mitochondrial gene expression after co-treatment with COFE and dorsomorphin compared to COFE or metformin alone. Data are presented as mean ± standard error (SE), *n* = 3. Statistical significance was determined using one-way ANOVA followed by Tukey’s post hoc test or unpaired two-tailed *t*-test, as appropriate. * *p* < 0.05, ** *p* < 0.01, *** *p* < 0.001 vs. blank control (BC); ## *p* < 0.01, ### *p* < 0.001 vs. 1% COFE; δ *p* < 0.05, δδ *p* < 0.01, δδδ *p* < 0.001 vs. metformin.

To validate the transcriptomic prediction of mitochondrial enhancement, AMPK activation and mitochondrial gene expression were assessed. Western blot analysis showed that COFE increased AMPK phosphorylation, with 1% COFE exhibiting effects comparable to those induced by 1 mM metformin (Figure 3a). Original western blot scans are available in Supplementary Files. In parallel, qPCR analysis demonstrated significant upregulation of mitochondrial respiratory chain genes, including *NDUFB8* and *NDUFA4L2* (Complex I), *SDHB* (Complex II), *UQCRHL* (Complex III), *COX6B2* (Complex IV), and *ATP6V0B* (proton transport, associated with Complex V) (Figure 3c). Additionally, the expression of *PGC-1α* and *TFAM*, two master regulators of mitochondrial biogenesis and transcription, was markedly elevated.

To determine the involvement of AMPK signaling in these effects, the AMPK inhibitor dorsomorphin was co-administered. As shown in Figure 3b,d, dorsomorphin significantly attenuated COFE-induced AMPK phosphorylation and suppressed the upregulation of representative mitochondrial genes, including *PGC-1α*, *TFAM*, and *COX6B2*. These results demonstrate that COFE regulates mitochondrial gene expression in an AMPK-dependent manner.

**Figure 4:**
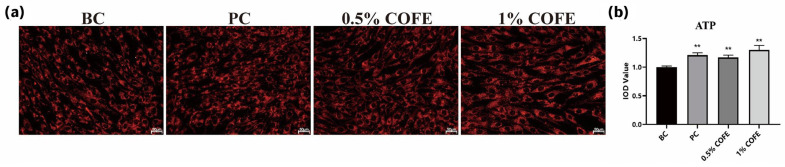
Effect of COFE on ATP synthesis in fibroblasts. (**a**) Representative fluorescence images of ATP synthesis in fibroblasts treated with positive control (PC), 0.5% COFE, and 1% COFE. Scale bar: 50 μm. (**b**) Quantification of intracellular ATP levels based on integrated optical density (IOD). Data are expressed as mean ± standard deviation (SD). The baseline control (BC) group received no treatment. Statistical analysis was performed using unpaired two-tailed *t*-test. ** *p* < 0.01 vs. BC group.

To evaluate whether these transcriptional and signaling changes translated into enhanced cellular bioenergetics, intracellular ATP levels were measured using TMRM fluorescence staining. COFE treatment resulted in a significant, dose-dependent increase in ATP-related fluorescence intensity, with increases of 17.00% and 30.00% observed at 0.5% and 1.0% concentrations, respectively (*p* < 0.01 vs. BC; Figure 4a,b). These findings confirm that COFE enhances mitochondrial bioenergetic capacity at both molecular and functional levels.

### 3.3. COFE Exhibits Broader and Less Inflammatory Transcriptional Responses than Retinol

RNA-seq analysis revealed distinct transcriptional profiles in response to retinol and COFE treatment. Compared to the blank control, retinol upregulated 136 genes and downregulated 114 genes, while COFE modulated a significantly broader gene set, with 1239 genes upregulated and 1205 downregulated (Figure 2a–c).

Gene Ontology (GO) enrichment analysis showed that retinol predominantly affected pathways related to retinoic acid metabolism and mitochondrial matrix organization (Figure 2d). KEGG pathway analysis further revealed enrichment in oxidative phosphorylation and glutathione metabolism (Figure 2e). At the gene level, retinol upregulated several retinoid metabolism-associated genes, including *CYP26B1*, *CRABP2*, *DHRS3*, and *RARA*, as well as mitochondrial genes such as *COX8A*, *NDUFS7*, and *NDUFA2*.

However, retinol also induced the expression of multiple inflammation-related genes and pathways, notably those involved in NOD-like and RIG-I-like receptor signaling (CCL2, NFKB1), suggesting a potential pro-inflammatory response.

In contrast, COFE treatment activated a broader array of transcriptional programs, particularly those associated with extracellular matrix organization, mitochondrial function, hormone metabolism, and immune modulation (Figure 2f,g). Representative genes included *COX6B2*, *ATP6V0B*, and *NDUFA4L2*, which contribute to oxidative phosphorylation, alongside robust upregulation of ECM-related genes.

Collectively, these findings suggest that while both COFE and retinol target mitochondrial and ECM pathways, COFE achieves these effects with broader transcriptomic engagement and reduced activation of inflammatory signaling cascades. This mechanistic distinction may reflect a more favorable safety profile for COFE in anti-aging applications, especially under oxidative or inflammatory stress conditions.

### 3.4. COFE Mitigates UVA-Induced Oxidative Stress and Restores Mitochondrial Function in Fibroblasts

**Figure 5:**
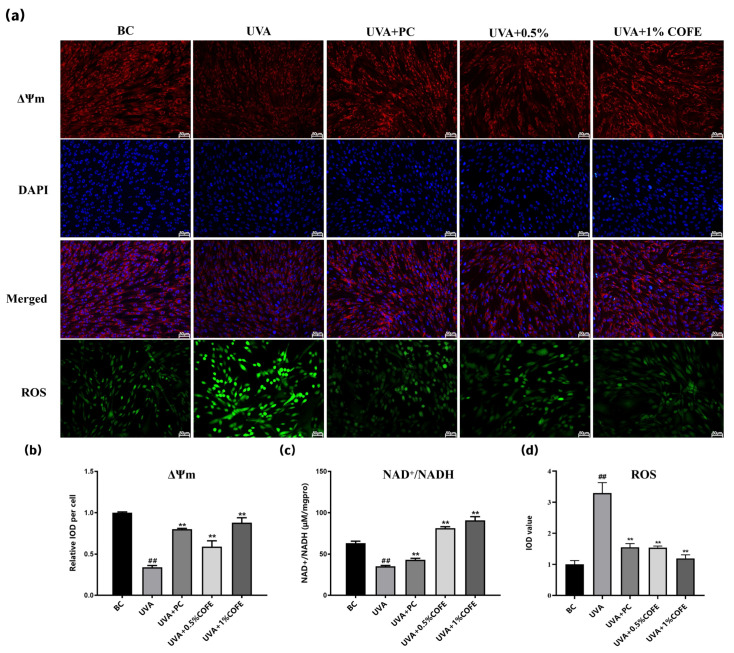
COFE mitigates UVA-induced mitochondrial dysfunction and oxidative stress in fibroblasts. (**a**) Representative immunofluorescence images showing mitochondrial membrane potential (ΔΨm, red) and nuclear staining (DAPI, blue) in fibroblasts after UVA irradiation, with or without treatment with *Cornus officinalis* fruit extract (COFE, 0.5% and 1.0%). Quercetin (300 nM) was used as a positive control. Merged images display overlays of ΔΨm and DAPI. Intracellular ROS was measured separately by DCFH-DA staining (green). Vitamin E (0.1%) was used as a positive control. Scale bars = 50 μm for all panels. (**b**) Quantification of ΔΨm levels based on integrated optical density (IOD) per cell. (**c**) Measurement of intracellular NAD^+^/NADH ratio. (**d**) Quantification of intracellular ROS levels based on IOD of DCF fluorescence. Data are presented as mean ± standard deviation (SD), *n* = 3. Statistical analysis was performed using unpaired two-tailed *t*-test. ** *p* < 0.01 vs. UVA group; ## *p* < 0.01 vs. baseline control (BC, untreated and non-irradiated).

To assess the protective effects of COFE on mitochondrial function under oxidative stress, intracellular ROS levels were measured in UVA-irradiated fibroblasts using DCFH-DA fluorescence staining. As shown in Figure 5a,d, UVA exposure markedly increased ROS levels compared to the blank control (BC), as indicated by significantly elevated IOD values (*p* < 0.01). Treatment with 0.5% and 1% COFE significantly reduced ROS accumulation relative to the negative control (UVA group), with inhibition rates of 53.19% and 63.83%, respectively (*p* < 0.01). These findings demonstrate COFE’s antioxidant capacity in mitigating UVA-induced oxidative stress in fibroblasts.

To assess the protective effects of COFE on mitochondrial function under oxidative stress, human dermal fibroblasts were exposed to UVA irradiation and treated with COFE at two concentrations (0.5% and 1.0%, *v*/*v*). Mitochondrial membrane potential (ΔΨm) was evaluated by TMRM staining (Figure 5a), with quantification showing a significant reduction in ΔΨm in UVA-exposed cells compared to the blank control (BC) (Figure 5b). Treatment with COFE significantly restored ΔΨm in a concentration-dependent manner, with increases of 73.53% and 158.82% at 0.5% and 1.0% COFE, respectively (*p* < 0.01 vs. UVA).

Redox balance was assessed by measuring the intracellular NAD^+^/NADH ratio normalized to total protein content (Figure 5c). UVA irradiation markedly suppressed the NAD^+^/NADH ratio compared to the control group (*p* < 0.01), indicating impaired mitochondrial redox status. COFE treatment significantly reversed this effect, with the NAD^+^/NADH ratio elevated by 131.54% and 158.71% at 0.5% and 1.0% concentrations, respectively (*p* < 0.01 vs. UVA). These findings suggest that COFE enhances mitochondrial function and restores redox homeostasis under UVA-induced oxidative stress.

### 3.5. COFE Promotes Epidermal Repair and Suppresses Inflammation in a 3D Epidermal Model

**Figure 6:**
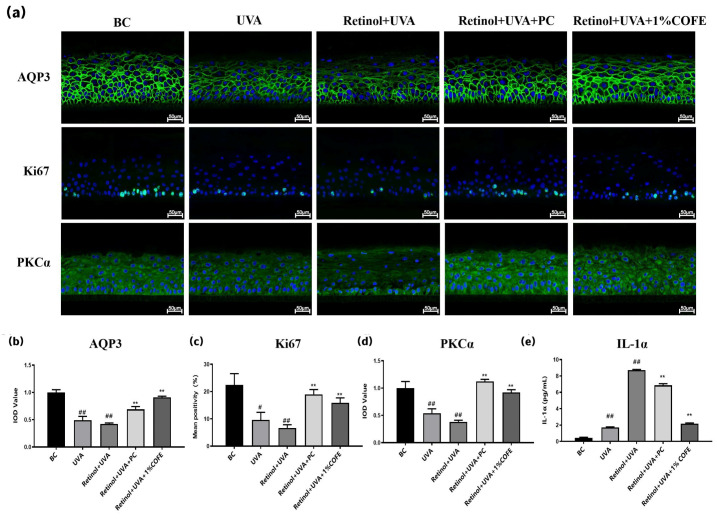
COFE mitigates retinol-induced phototoxicity under UVA exposure in the EpiKutis^®^ 3D epidermal model. (**a**) Immunofluorescence staining of AQP3, Ki67, and PKCα in EpiKutis^®^ models from five groups: baseline control (BC), UVA alone, Retinol + UVA, Retinol + UVA + 1% COFE, and Retinol + UVA + positive control Vitamin E (PC). Green: target proteins; blue (DAPI): nuclei. Scale bar = 50 μm. (**b**–**d**) Quantification of AQP3, Ki67, and PKCα expression by IOD or mean positivity (%). (**e**) IL-1α levels in supernatants measured by ELISA. Data are mean ± SD (*n* = 3). Statistical analysis was performed using unpaired two-tailed *t*-test. # *p* < 0.05, ## *p* < 0.01 vs. BC; ** *p* < 0.01 vs. Retinol + UVA.

As shown in Figure 6a–e, combined treatment with retinol and UVA significantly impaired epidermal function, as evidenced by reduced expression of AQP3, Ki67, and PKCα, along with markedly elevated IL-1α secretion. These alterations indicate diminished skin hydration, suppressed keratinocyte proliferation, and increased inflammation. Treatment with 1% COFE effectively reversed these detrimental effects.

Quantitatively, IL-1α levels were significantly reduced by 75.23% compared to the Retinol + UVA group (Figure 6e). The expression of AQP3 increased by 116.67%, indicating improved hydration (Figure 6b). Ki67 positivity increased by 138.05%, reflecting enhanced keratinocyte proliferation (Figure 6c), and PKCα expression rose by 142.11%, suggesting restoration of epidermal signaling involved in barrier repair and differentiation (Figure 6d). These results demonstrate that COFE markedly mitigates retinol- and UVA-induced epidermal dysfunction by promoting hydration and proliferation, enhancing barrier-associated signaling, and exerting anti-inflammatory activity in a reconstructed human epidermal model.

### 3.6. Eye Cream Formulated with COFE Demonstrated Periorbital Anti-Aging Effects

**Figure 7:**
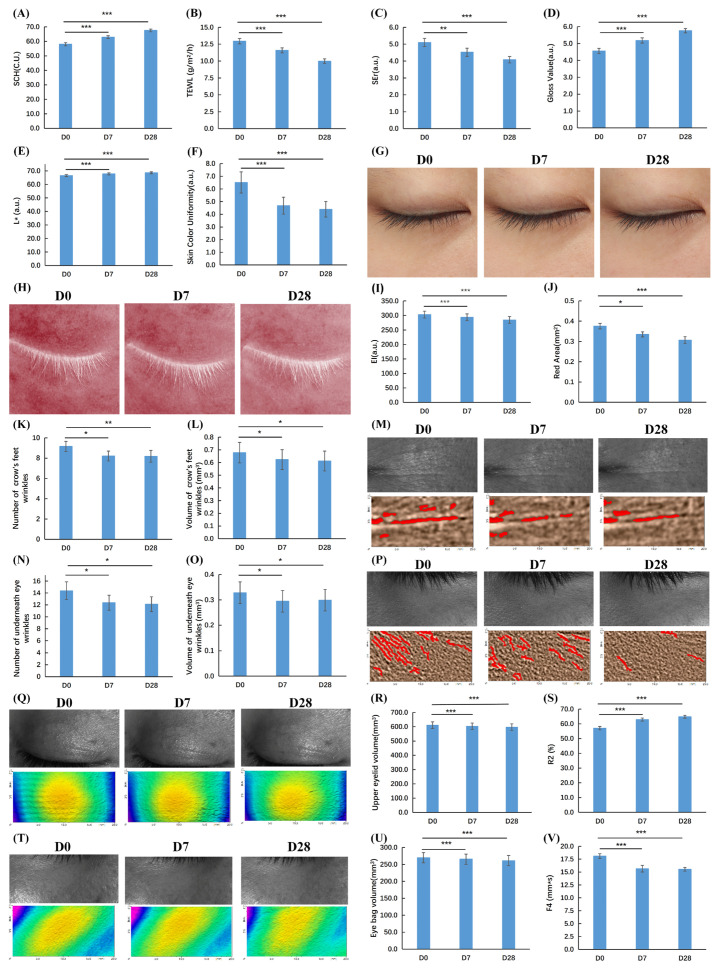
Instrumental evaluation of the in vivo efficacy of the eye cream formulated with 1% COFE. (**A**): Stratum corneum hydration; (**B**): Transepidermal water loss (TEWL); (**C**): Skin roughness (SEr); (**D**): Skin glossiness; (**E**): Skin brightness (L* value); (**F**): Skin tone evenness; (**G**): Representative case of glossiness improvement; (**H**): Representative case of erythema reduction; (**I**): Lower-eyelid hemoglobin index; (**J**): Skin redness area; (**K**): Crow’s feet wrinkle count; (**L**): Crow’s feet wrinkle volume; (**M**): Representative improvement of crow’s feet; (**N**): Under-eye fine line count; (**O**): Under-eye fine line volume; (**P**): Representative improvement of under-eye wrinkles; (**Q**): Representative improvement of upper eyelid; (**R**): Upper-eyelid volume; (**S**): Skin elasticity (R2); (**T**): Representative improvement of under-eye bags; (**U**): Under-eye bag volume; (**V**): Skin firmness (F4). Data are presented as mean ± SEM. Compared with D0, “*” indicates *p* < 0.05, “**” indicates *p* < 0.01, and “***” indicates *p* < 0.001. Participants: *n* = 34.

Comprehensive clinical outcomes are summarized in Table 2, with representative improvement cases shown in Figure 7. Consistent with the quantitative data, statistically significant improvements were observed across all assessed periorbital skin parameters over the 28-day intervention period (*p* < 0.05), indicating progressive enhancement of skin condition.

Relative to baseline (D0), stratum corneum hydration at the canthus increased by 8.39% at Day 7 (D7) and 16.44% at Day 28 (D28), while TEWL decreased by 10.51% and 22.87%, respectively, reflecting improved skin moisturization and barrier function. Skin surface texture showed continuous refinement, with roughness (SEr) reduced by 11.37% at D7 and 20.00% at D28. Periorbital redness was alleviated, as evidenced by reductions in the erythema index (3.12% at D7; 6.12% at D28) and red-area proportion (10.77% and 18.28%, respectively).

Parallel improvements were observed in skin tone and optical properties. Lower-eyelid brightness (L*) increased by 1.86% at D7 and 3.09% at D28, skin tone evenness improved by 28.11% and 32.57%, and skin glossiness increased by 13.60% and 26.32%, respectively, indicating a clearer and more radiant periorbital appearance. Wrinkle analysis revealed reductions in both wrinkle volume and count. Crow’s feet wrinkle volume decreased by 8.12% (D7) and 9.76% (D28), with wrinkle number reduced by 10.27% and 10.60%. Under-eye fine line volume decreased by 10.27% at D7 and 9.02% at D28, while line count declined by 13.91% and 15.72%, respectively.

Biomechanical skin properties also improved significantly. Skin elasticity (R2) increased by 10.19% at D7 and 13.56% at D28, while firmness (F4) increased by 13.48% and 14.20%, reflecting enhanced dermal resilience and structural support. In addition, volumetric contour analysis showed reductions in upper-eyelid volume (1.30% at D7; 2.26% at D28) and under-eye bag volume (1.58% and 3.17%), suggesting improvements in periorbital puffiness and tissue laxity.

Collectively, 28-day application of the eye cream resulted in broad and consistent improvements in hydration, barrier integrity, skin tone, redness, texture, wrinkle severity, biomechanical properties, and periorbital contour. These clinical benefits are consistent with the proposed role of COFE in enhancing mitochondrial bioenergetics via AMPK–OXPHOS-related pathways, as supported by the mechanistic findings of this study.

## 4. Discussion

This study demonstrates that a standardized *Cornus officinalis* fruit extract (COFE), derived from the Qinling Mountains, enhances mitochondrial bioenergetics and confers cytoprotective benefits under oxidative and environmental stress. The phytochemical richness of COFE—particularly its iridoid glycosides and phenolic constituents—is consistent with prior reports describing *C. officinalis* as an antioxidant and mitochondrial-regulatory botanical [30,33]. Here, we provide the first evidence linking COFE to AMPK-associated mitochondrial functional activation in skin-relevant systems, highlighting its potential as a bioenergetic modulator in cutaneous aging.

ATP is essential for sustaining redox homeostasis, barrier integrity, and the high metabolic demands of skin tissue. Age-associated mitochondrial dysfunction leads to reduced dermal ATP production, an effect further exacerbated by UV radiation and other environmental stressors [13,34,35,36]. These alterations impair oxidative phosphorylation (OXPHOS), the primary ATP-generating pathway in skin cells, accelerating visible aging and compromising tissue repair capacity [37]. Identifying natural bioactives that can enhance mitochondrial ATP production through defined biochemical pathways therefore represents an important direction in dermatologic pharmacology. Despite growing interest in botanical actives, few have been mechanistically validated in this context.

**Figure 8:**
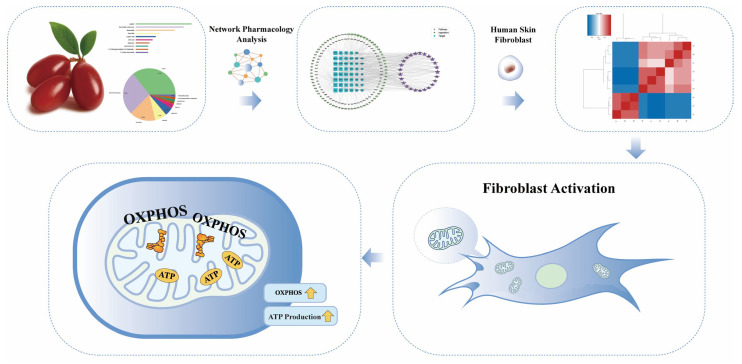
Proposed mechanism and experimental strategy for evaluating the mitochondrial bioactivation effects of *Cornus officinalis* fruit extract (COFE).

Our multi-omics workflow—including LC–MS metabolite profiling, network pharmacology, and RNA sequencing—revealed that COFE upregulates pathways associated with OXPHOS, redox regulation, and ECM remodeling. qPCR validation confirmed increased expression of representative genes across mitochondrial respiratory complexes I–V (e.g., *NDUFB8*, *SDHB*, *UQCRCL*, *COX6B2*, and *ATP6V0B*), accompanied by enhanced ATP-associated signal intensity in unstressed fibroblasts. Under UVA-induced oxidative stress, COFE restored mitochondrial membrane potential (ΔΨm), elevated the NAD^+^/NADH ratio, and reduced intracellular ROS accumulation. Together, these convergent and relative functional readouts—rather than absolute ATP quantification—indicate preservation and enhancement of mitochondrial functional performance and redox balance under stress conditions. A schematic summary of the experimental workflow and proposed mechanism of action is presented in Figure 8.

Mechanistically, COFE’s mitochondrial activation was associated with AMPK activation, as evidenced by increased phosphorylation of AMPK (Thr172) and the loss of COFE-induced ATP enhancement in the presence of the AMPK inhibitor dorsomorphin. These data support a role for AMPK as an upstream regulatory node governing mitochondrial transcriptional programming, through which COFE modulates bioenergetic capacity. Given that downstream functional endpoints such as ATP levels, ΔΨm, and NAD^+^/NADH ratio reflect integrated mitochondrial outcomes influenced by multiple convergent pathways, AMPK activation is best interpreted as a critical regulatory contributor rather than the sole determinant of all observed functional effects. Consistent with AMPK’s recognized role in metabolic homeostasis, stress adaptation, and aging biology [38,39], these findings position COFE as a promising naturally derived AMPK modulator with relevance for cutaneous health.

To contextualize COFE’s performance, we compared its transcriptomic effects with those of retinol, a benchmark anti-aging active with well-characterized limitations related to irritancy and photoreactivity [40,41]. Both actives upregulated OXPHOS- and ECM-related genes, suggesting mechanistic convergence in pathways linked to dermal rejuvenation. Importantly, in a 3D epidermal model preconditioned with retinol and subsequently exposed to UVA to mimic stress under real-world retinoid use, COFE reduced IL-1α secretion and restored markers of hydration (AQP3), proliferation (Ki67), and barrier integrity (PKCα), indicating that COFE may mitigate the photo-oxidative fragility often associated with retinoid-treated skin.

Crucially, the mechanistic benefits of COFE were accompanied by measurable improvements in vivo. In a 28-day clinical study, a 1% COFE eye cream produced statistically significant improvements across hydration, barrier function, redness, skin tone, texture, wrinkle severity, elasticity, and periorbital contour. However, in the absence of a vehicle-controlled or comparator arm, the relative contribution of formulation excipients versus COFE itself cannot be definitively distinguished. Moreover, AMPK–OXPHOS pathway activation was not directly assessed in vivo, and therefore mechanistic conclusions derived from cellular models may not be directly extrapolated to human skin. The clinical findings should thus be interpreted as phenotypic and functional improvements consistent with, but not mechanistically proven to result from, the mitochondrial effects observed in vitro. These outcomes remain notable given the unique vulnerability of the periocular region, which is characterized by a thin epidermis, low lipid content, and heightened susceptibility to oxidative and mechanical stress.

This study has limitations. First, although multiple skin-relevant experimental models were employed, direct assessment of mitochondrial dynamics and long-term cellular fate was not performed. Functional mitochondrial flux analysis, senescence-associated markers (such as p16, p21, and SA-β-gal), and apoptosis-related endpoints were not evaluated and would provide valuable insight into aging-associated cellular outcomes. Second, although transcriptional regulators related to mitochondrial maintenance (e.g., PGC-1α and TFAM) were modulated, direct evaluation of mitochondrial biogenesis—such as measurements of mitochondrial mass, mitochondrial DNA copy number, or ultrastructural morphology—was not conducted, and such analyses would be required to substantiate biogenesis-related conclusions. These measurements therefore represent important directions for future investigation.

Third, while AMPK involvement was supported at the signaling and transcriptional levels, downstream mitochondrial functional endpoints (including ATP-associated signal intensity, ΔΨm, and NAD^+^/NADH ratio) were not assessed under pharmacological AMPK inhibition. Incorporation of AMPK-inhibited functional assays would strengthen causal interpretation and represents an important avenue for future mechanistic validation. In addition, although fluorescence-based ATP assays are suitable for comparative live-cell analyses, orthogonal ATP quantification approaches (such as luminescence-based or chromatographic methods) would further enhance quantitative bioenergetic interpretation. Cell viability was evaluated using the MTT assay as a cytocompatibility screen; future studies incorporating viability assays less dependent on mitochondrial metabolism, including membrane integrity- or DNA content-based methods, would provide complementary information.

Finally, although the clinical evaluation provided translational relevance, larger-scale randomized, placebo-controlled, double-blind studies with extended follow-up durations will be required to more fully define COFE’s therapeutic potential and its comparative performance relative to established actives. Notwithstanding these limitations, the concordance between molecular, functional, tissue-level, and clinical observations supports the concept that COFE enhances mitochondrial functional resilience under oxidative stress.

## 5. Conclusions

In conclusion, COFE functions as an AMPK-associated mitochondrial bioenergetic modulator that enhances mitochondrial functional performance, redox homeostasis, and epidermal resilience under oxidative stress in skin-relevant experimental models. The concordance between mechanistic findings and observed clinical improvements provides a rationale for further controlled and mechanistically informed studies evaluating COFE as a dermocosmetic ingredient targeting mitochondrial dysfunction associated with skin aging.

## Figures and Tables

**Table 1:** Primer sequences used for qRT-PCR analysis of mitochondrial and metabolic genes in human dermal fibroblasts.

Gene Symbol	Sequences of Primer
H-ATP6V0B-F	CCATCGGAACTACCATGCAGG
H-ATP6V0B-R	TCCACAGAAGAGGTTAGACAGG
H-UQCRHL-F	AATGTGTAAAGGCCCGGGAG
H-UQCRHL-R	GTTTGTGGGCCACGCAATG
H-NDUFA4L2-F	ATGATCGGCTTAATCTGCCTG
H-NDUFA4L2-R	TCCGGGTTGTTCTTTCTGTCC
H-COX6B2-F	TTCCTGGACTACCACCGCT
H-COX6B2-R	CGGCGAAAATCCCGTTCTTG
H-PGC-1α-F	TCTGAGTCTGTATGGAGTGACAT
H-PGC-1α-R	CCAAGTCGTTCACATCTAGTTCA
H-TFAM-F	ATGGCGTTTCTCCGAAGCAT
H-TFAM-R	TCCGCCCTATAAGCATCTTGA
H-NDUFB8-F	ACAGGAACCGTGTGGATACAT
H-NDUFB8-R	CCCCACCCAGCACATGAAT
H-SDHB-F	GACACCAACCTCAATAAGGTCTC
H-SDHB-R	GGCTCAATGGATTTGTACTGTGC

Primers were synthesized by Tsingke Biotech. “F” and “R” indicate forward and reverse primers, respectively.

**Table 2:** A summary of clinical results of the 1% COFE eye cream.

Parameters	Baseline (D0)	D7		D28	
		Mean ± SEM	Improvement	Mean ± SEM	Improvement
Stratum corneum hydration	58.03 ± 1.14	62.90 ± 1.00	8.39% ***	67.57 ± 0.91	16.44% ***
TEWL	12.94 ± 0.39	11.58 ± 0.36	−10.51% ***	9.98 ± 0.34	−22.87% ***
SEr	5.10 ± 0.24	4.52 ± 0.24	−11.37% **	4.08 ± 0.19	−20.00% ***
Erythema Index, EI	302.91 ± 11.65	293.46 ± 11.57	−3.12% ***	284.38 ± 11.72	−6.12% ***
Red Area	0.3759 ± 0.0133	0.3354 ± 0.0127	−10.77% *	0.3072 ± 0.0167	−18.28% ***
Gloss Value	4.56 ± 0.16	5.18 ± 0.15	13.60% ***	5.76 ± 0.13	26.32% ***
Skin Color Uniformity	6.51 ± 0.83	4.68 ± 0.67	−28.11% ***	4.39 ± 0.61	−32.57% ***
Skin brightness L*	66.56 ± 0.75	67.80 ± 0.76	1.86% ***	68.62 ± 0.75	3.09% ***
Number of crow’s feet wrinkles	9.15 ± 0.51	8.21 ± 0.48	−10.27% *	8.18 ± 0.58	−10.60% **
Volume of crow’s feet wrinkles	0.6783 ± 0.0811	0.6232 ± 0.0783	−8.12% *	0.6121 ± 0.0779	−9.76% *
Number of underneath eye wrinkles	14.38 ± 1.49	12.38 ± 1.25	−13.91% *	12.12 ± 1.24	−15.72% *
Volume of underneath eye wrinkles	0.3282 ± 0.0426	0.2945 ± 0.0426	−10.27% *	0.2986 ± 0.0423	−9.02% *
R2	0.5709 ± 0.0114	0.6291 ± 0.0108	10.19% ***	0.6483 ± 0.0099	13.56% ***
F4	18.10 ± 0.46	15.66 ± 0.65	−13.48% ***	15.53 ± 0.37	−14.20% ***
Upper eyelid volume	610.96 ± 23.28	603.02 ± 22.75	−1.30% ***	597.18 ± 22.68	−2.26% ***
Eye bag volume	269.67 ± 15.06	265.41 ± 15.11	−1.58% ***	261.11 ± 15.07	−3.17% ***

Compared with D0, “*” indicates *p* < 0.05, “**” indicates *p* < 0.01, and “***” indicates *p* < 0.001. Participants: *n* = 34.

## Data Availability

All data generated or analyzed during this study are included in this article. Further enquiries can be directed to the corresponding authors.

## Supplementary Materials

The original western blotting scans can be downloaded at: https://www.mdpi.com/article/10.3390/biomedicines14020403/s1.

## Abbreviations

The following abbreviations are used in this manuscript:

COFE	*Cornus officinalis* fruit extract
ECM	Extracellular matrix
PBS	Phosphate-buffered saline
AMPK	AMP-activated protein kinase
ΔΨm	Mitochondrial membrane potential
IOD	Integrated optical density
qPCR	Quantitative real-time polymerase chain reaction
ROS	Reactive oxygen species
GO	Gene ontology
OXPHOS	Oxidative phosphorylation
DEGs	Differentially expressed genes
NAD^+^/NADH	Nicotinamide adenine dinucleotide (oxidized/reduced form)
TFAM	Mitochondrial transcription factor A
PGC-1α	Peroxisome proliferator-activated receptor gamma coactivator 1-alpha
DCFH-DA	2′,7′-Dichlorodihydrofluorescein diacetate
