# Supplementary material for: Cornus officinalis Fruit Extract as an AMPK-Associated Mitochondrial Bioenergetic Modulator in Skin Aging Models
Source: Biomedicines. 2026 Feb 10;14(2):403. doi: 10.3390/biomedicines14020403 (PMC12938615; doi:10.3390/biomedicines14020403)

Repeat 1:

AMPK-1: Only the first set of bands belongs to this experiment. The other two sets on the same membrane are from other experiments run at the same time

Correct lane order: BC, COFE 0.5%, COFE 1%, Metformin

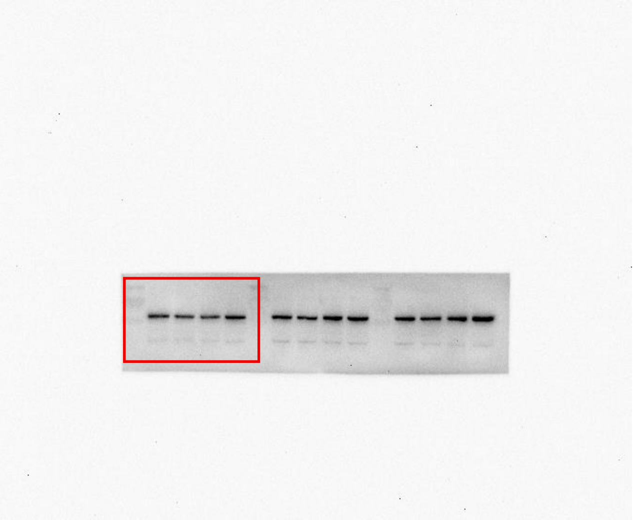

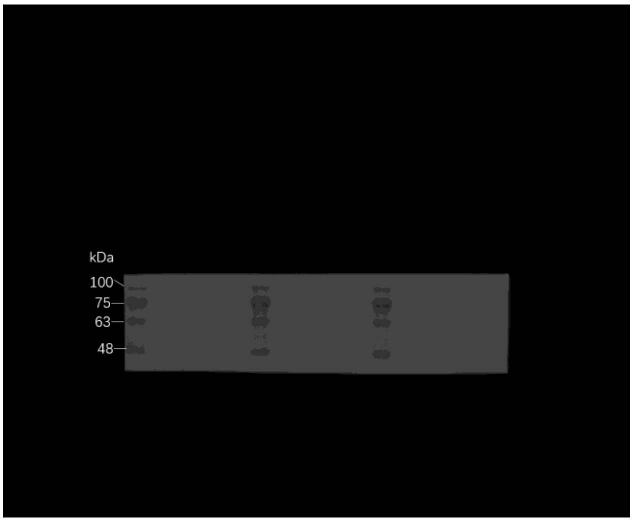

P-AMPK-1: Same situation as AMPK-1 plus one issue: the membrane was placed upside down during exposure. Therefore, the image must be horizontally flipped (left–right flipped)

After flipping, the correct order is: BC, COFE 0.5%, COFE 1%, Metformin

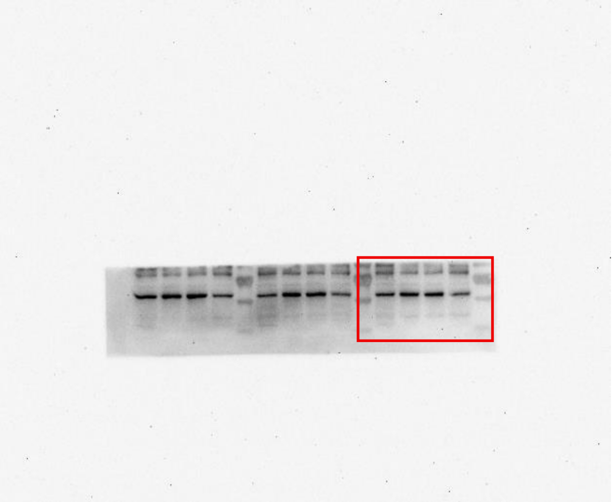

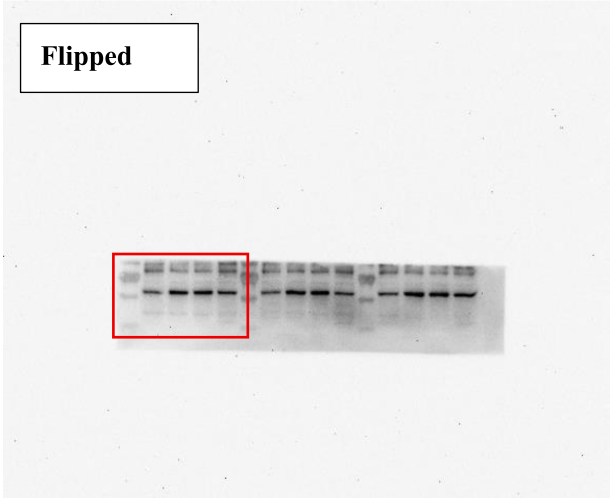

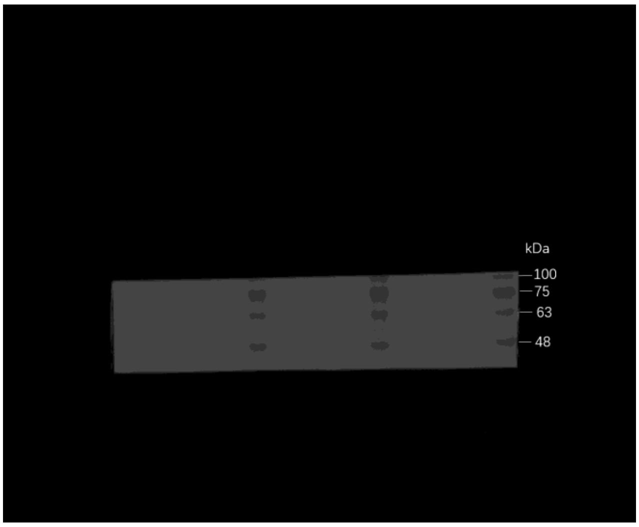

Loading control protein β-actin: Same issue as p-AMPK: Membrane upside down. Must be left–right flipped. After flipping, lane order is: BC, COFE 0.5%, COFE 1%, Metformin

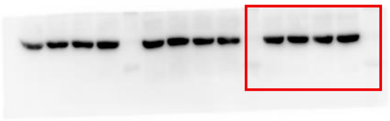

Flipped

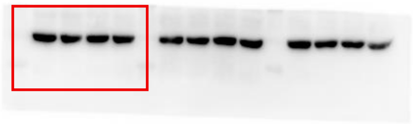

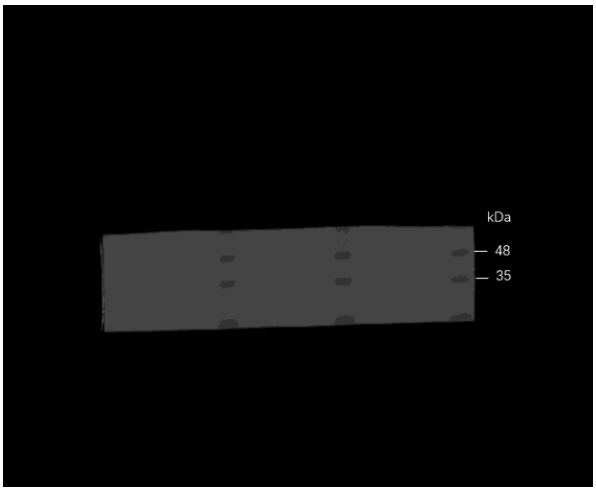

Repeat 2

AMPK-2:

Lane order: BC, COFE 0.5%, COFE 1%, Metformin

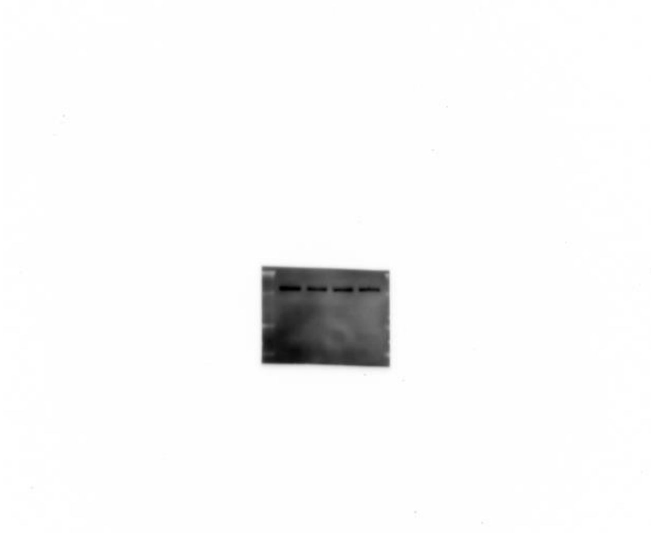

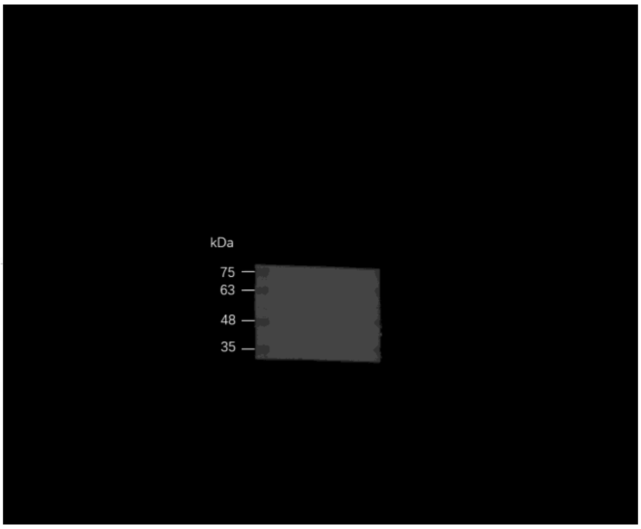

P-AMPK-2:

Lane order: BC, COFE 0.5%, COFE 1%, Metformin

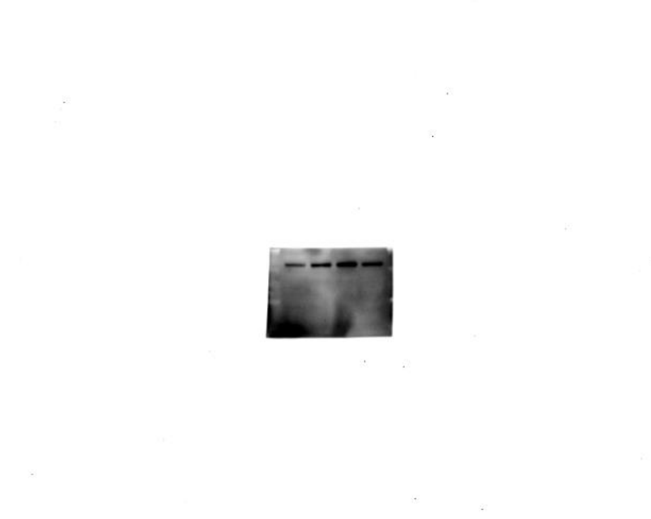

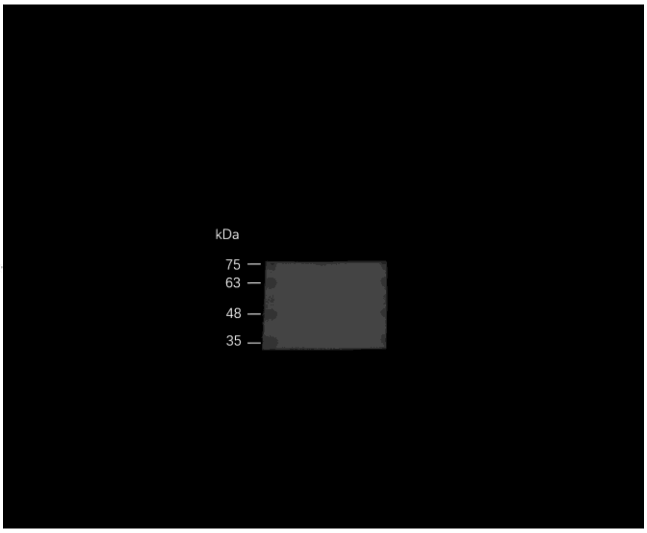

Loading control protein β-actin:

Lane order: BC, COFE 0.5%, COFE 1%, Metformin

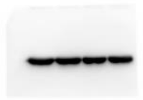

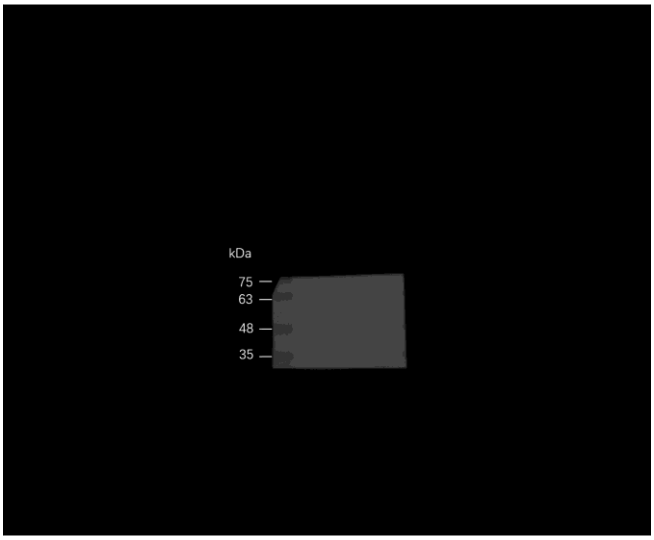

Repeat 3:
AMPK-3:
Lane order: BC, COFE 0.5%, COFE 1%, Metformin

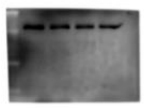

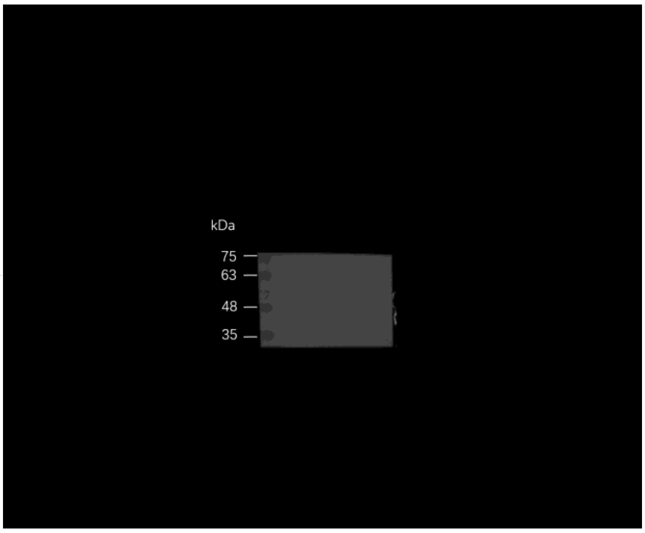

P-AMPK-3:
Lane order: BC, COFE 0.5%, COFE 1%, Metformin

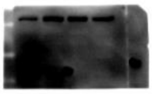

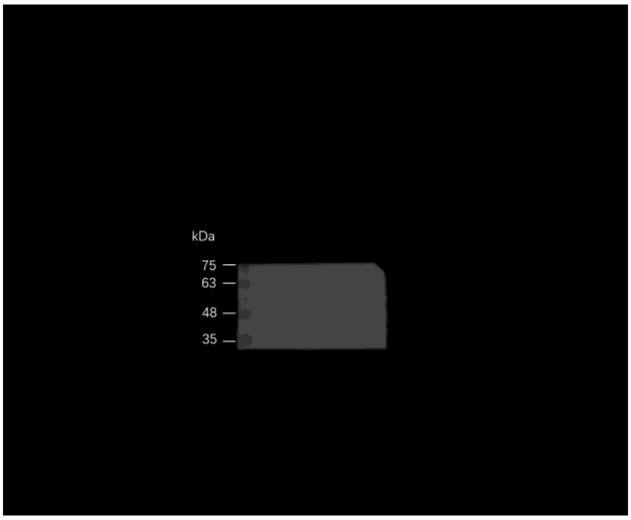

Loading control protein β-actin:

Lane order: BC, COFE 0.5%, COFE 1%, Metformin

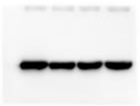

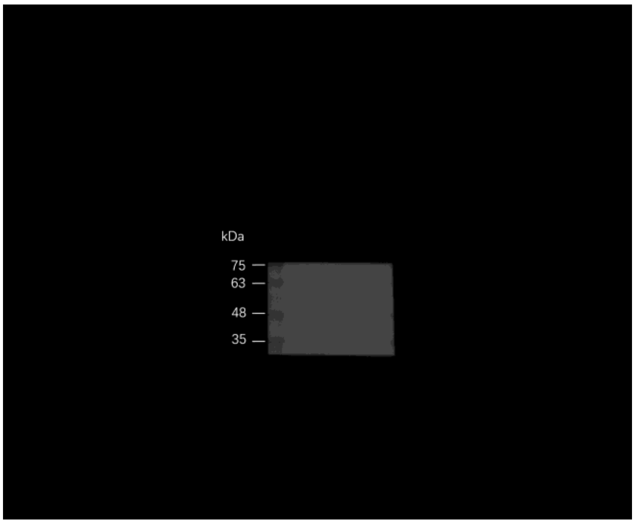

Supplement: Supplementary file 1 [file biomedicines-14-00403-s001.zip › Original Western blot clarification_1.pdf]
