# Supplementary material for: Cornus officinalis Fruit Extract as an AMPK-Associated Mitochondrial Bioenergetic Modulator in Skin Aging Models
Source: Biomedicines. 2026 Feb 10;14(2):403. doi: 10.3390/biomedicines14020403 (PMC12938615; doi:10.3390/biomedicines14020403)

Repeat 1:

AMPK-1:

Lane order: BC, COFE 1%, COFE 1% + Dorsomorphin, Metformin + Dorsomorphin

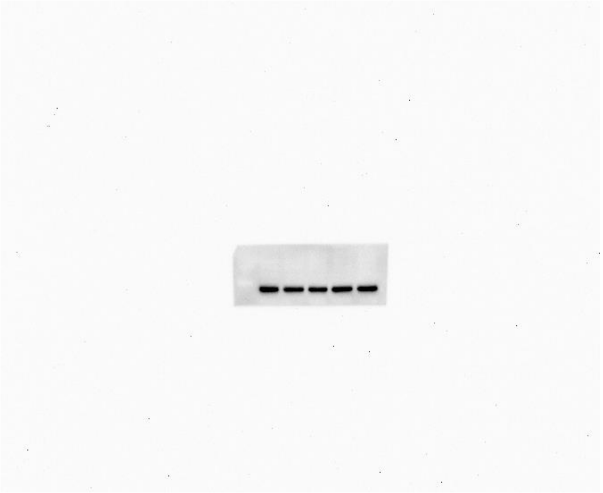

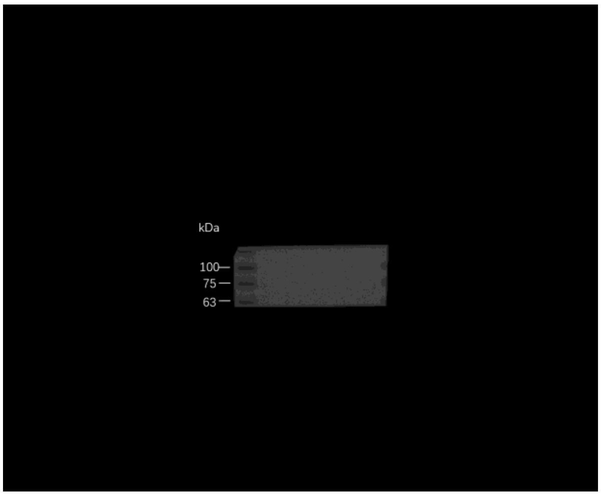

P-AMPK-1:

Lane order: BC, COFE 1%, COFE 1% + Dorsomorphin, Metformin + Dorsomorphin

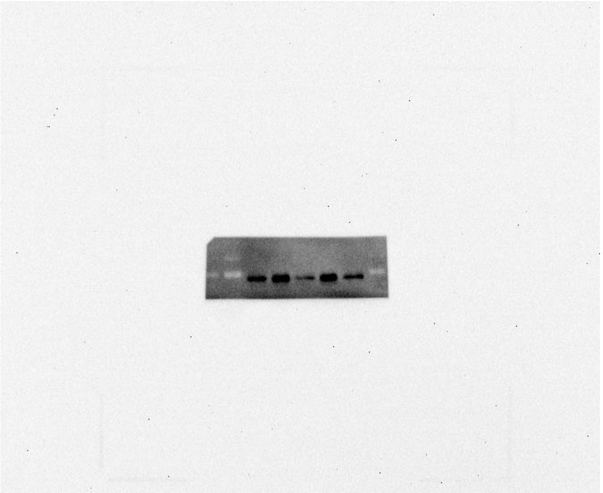

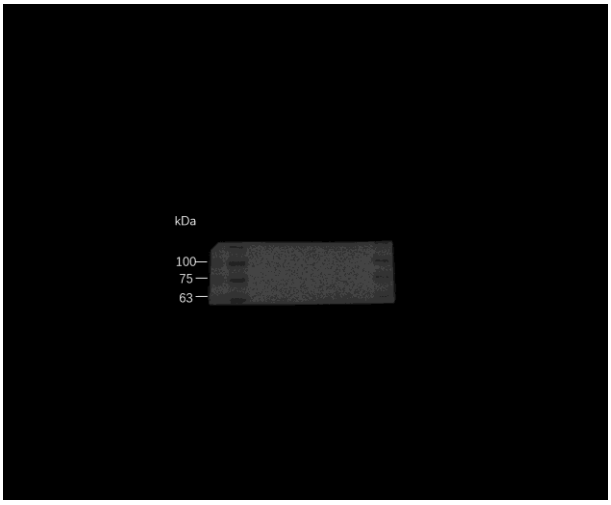

Loading control protein β-actin:

Lane order: BC, COFE 1%, COFE 1% + Dorsomorphin, Metformin + Dorsomorphin

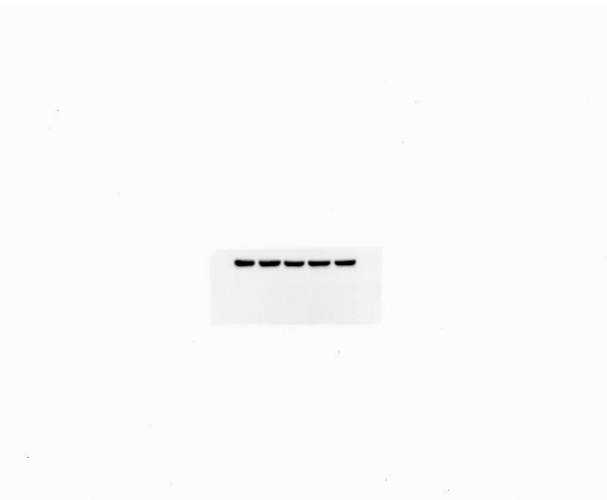

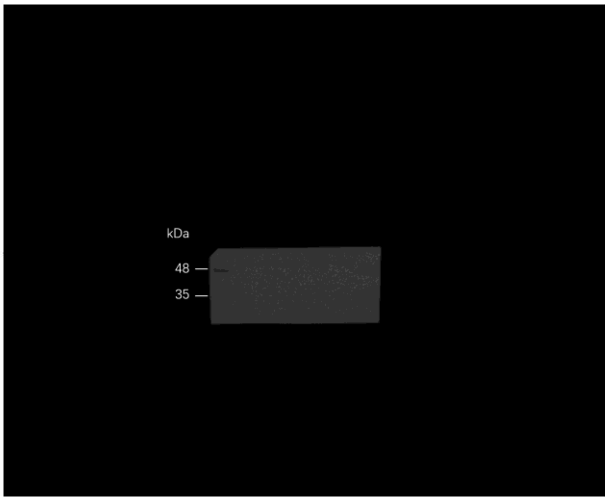

Repeat 2：

AMPK-2：

Lane order: BC, COFE 1%, COFE 1% + Dorsomorphin, Metformin + Dorsomorphin

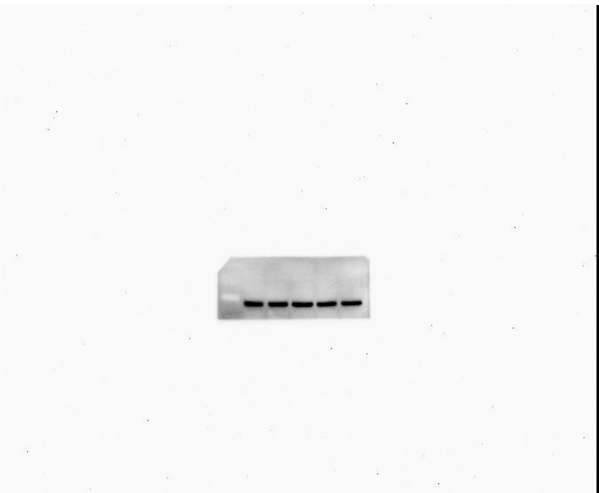

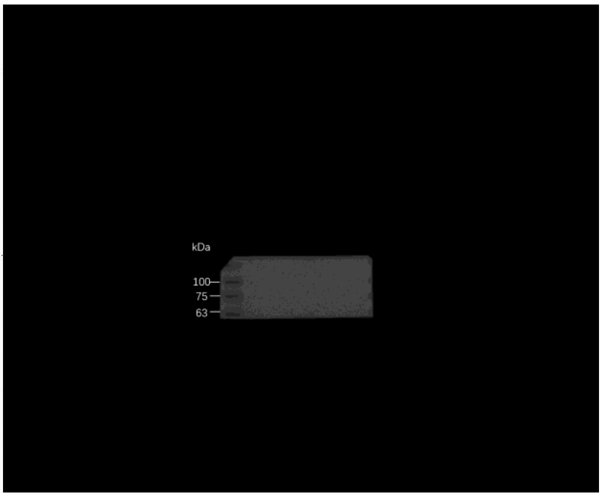

P-AMPK-2：

Lane order: BC, COFE 1%, COFE 1% + Dorsomorphin, Metformin + Dorsomorphin

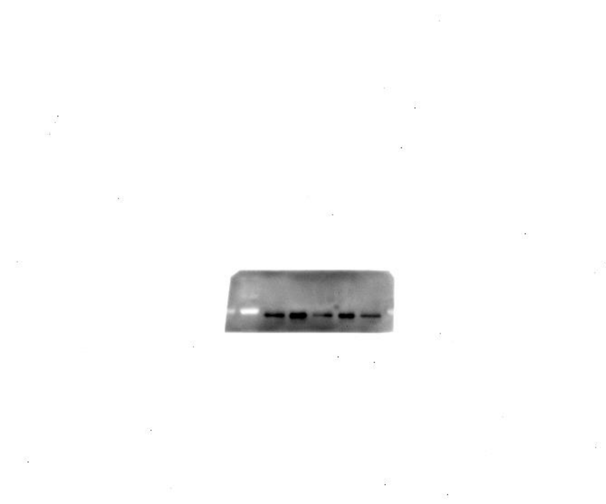

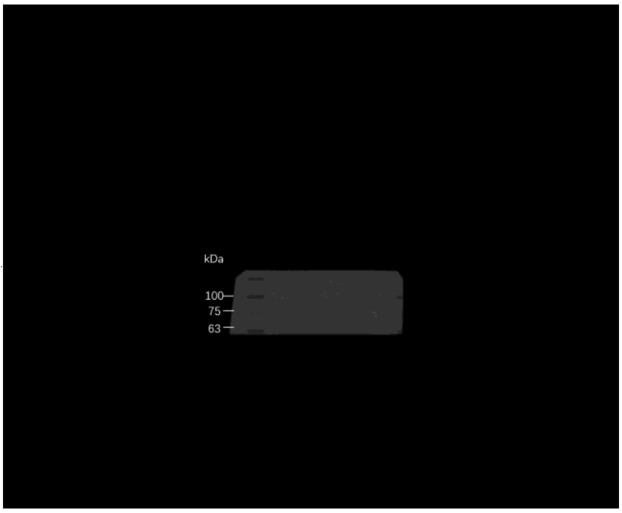

Loading control protein β-actin：

Lane order: BC, COFE 1%, COFE 1% + Dorsomorphin, Metformin + Dorsomorphin

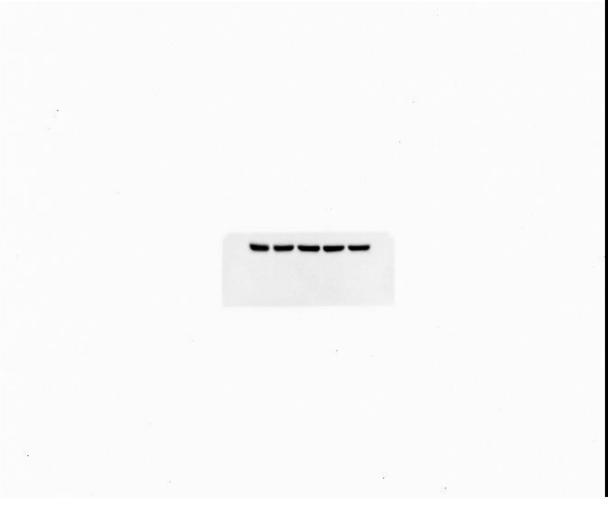

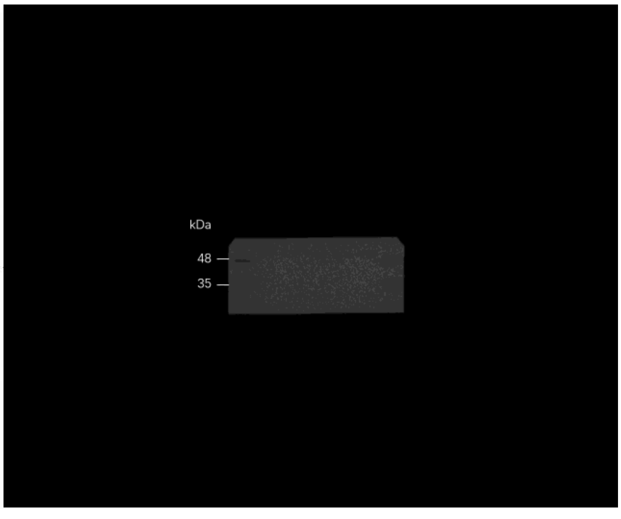

Repeat 3:

AMPK-3:

Lane order: BC, COFE 1%, COFE 1% + Dorsomorphin, Metformin + Dorsomorphin

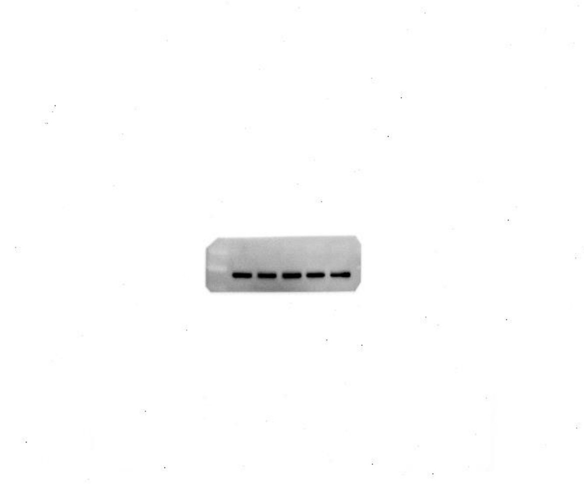

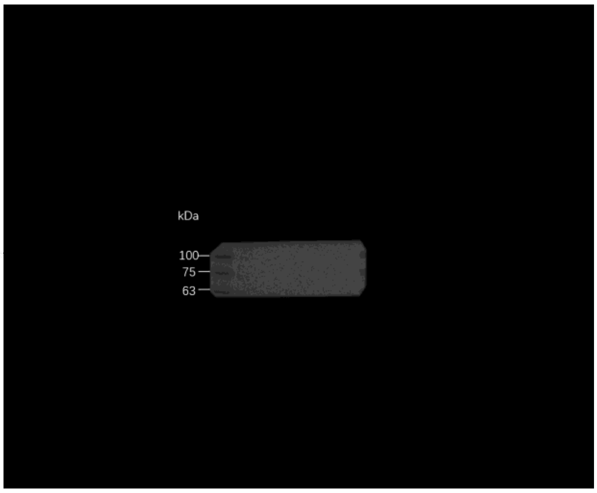

P-AMPK-3:

Lane order: BC, COFE 1%, COFE 1% + Dorsomorphin, Metformin + Dorsomorphin

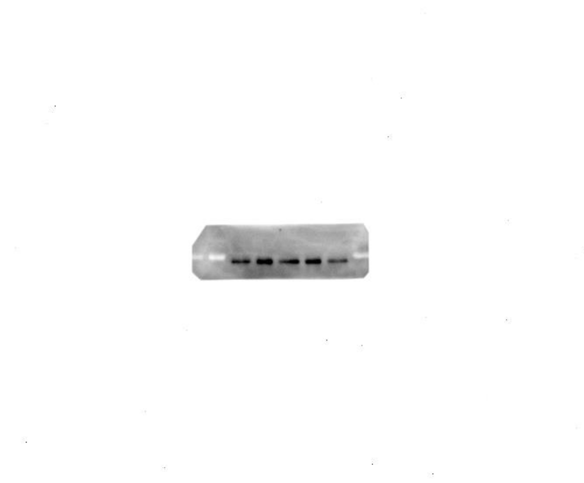

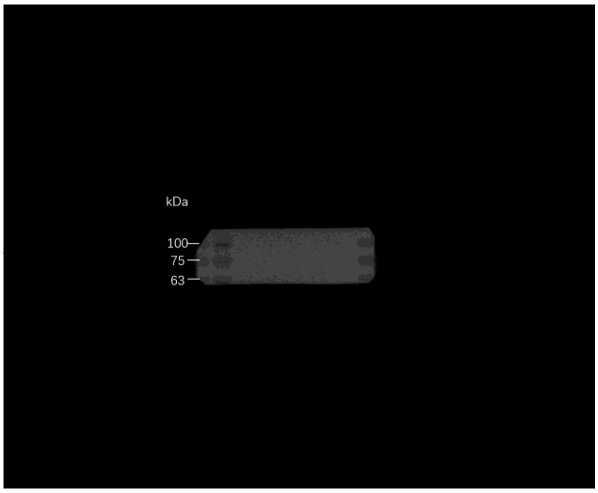

Loading control protein β-actin:

Lane order: BC, COFE 1%, COFE 1% + Dorsomorphin, Metformin + Dorsomorphin

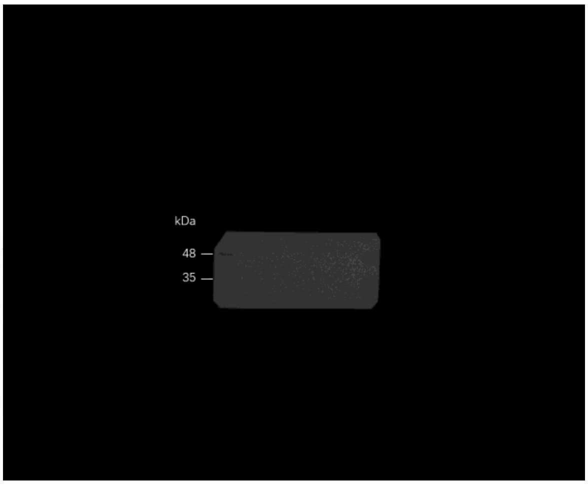

Supplement: Supplementary file 1 [file biomedicines-14-00403-s001.zip › Original Western blot clarification_2.pdf]
