# Supplementary figures and images for: Cornus officinalis Fruit Extract as an AMPK-Associated Mitochondrial Bioenergetic Modulator in Skin Aging Models
Source: Biomedicines. 2026 Feb 10;14(2):403. doi: 10.3390/biomedicines14020403 (PMC12938615; doi:10.3390/biomedicines14020403)

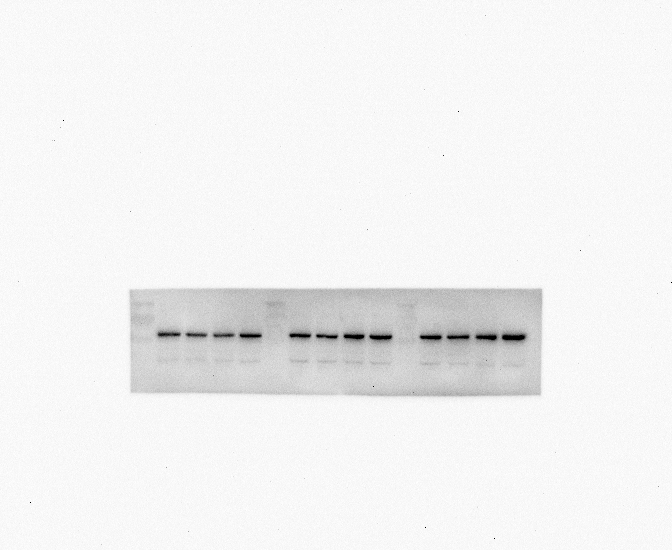

Supplement: Supplementary file 1 [file biomedicines-14-00403-s001.zip › a) WB original 1/AMPK-1_.tif]

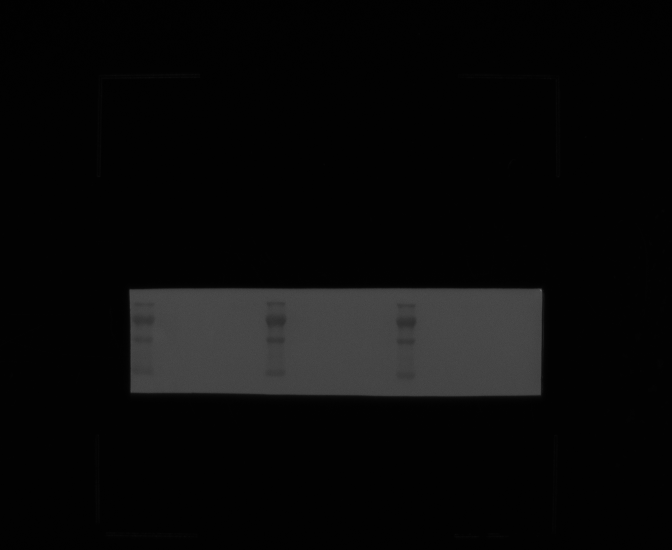

Supplement: Supplementary file 1 [file biomedicines-14-00403-s001.zip › a) WB original 1/AMPK-1_MARKER.tif]

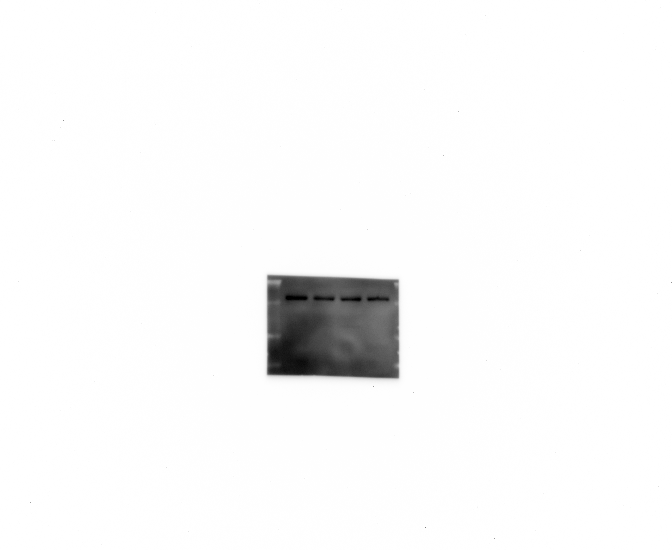

Supplement: Supplementary file 1 [file biomedicines-14-00403-s001.zip › a) WB original 1/AMPK-2.tif]

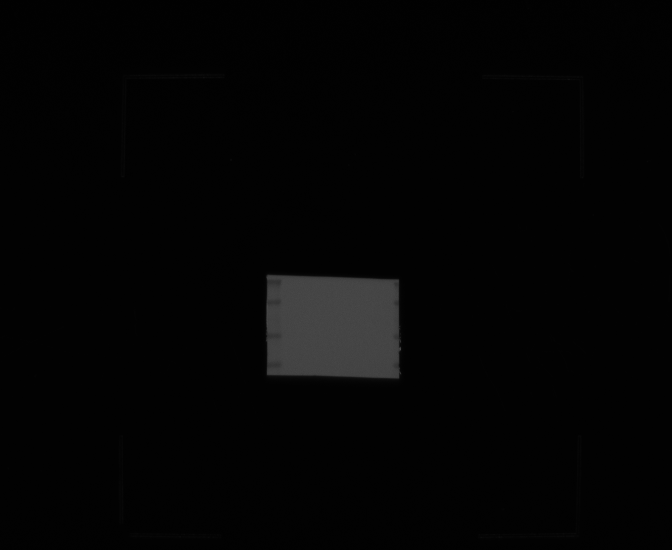

Supplement: Supplementary file 1 [file biomedicines-14-00403-s001.zip › a) WB original 1/AMPK-2_MARKER.tif]

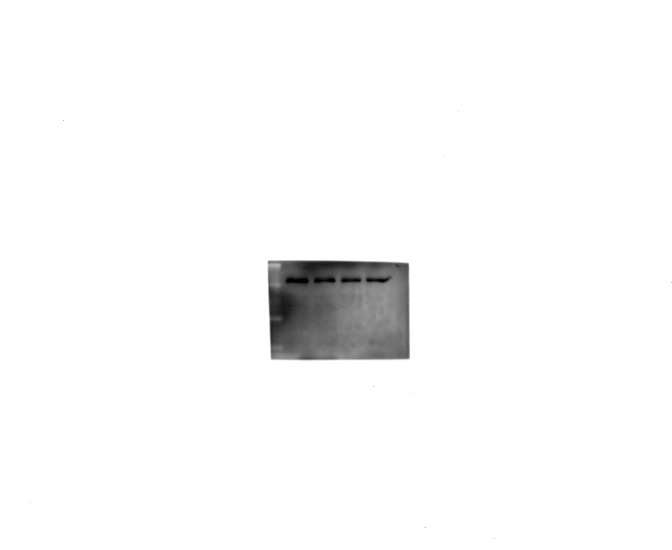

Supplement: Supplementary file 1 [file biomedicines-14-00403-s001.zip › a) WB original 1/AMPK-3.tif]

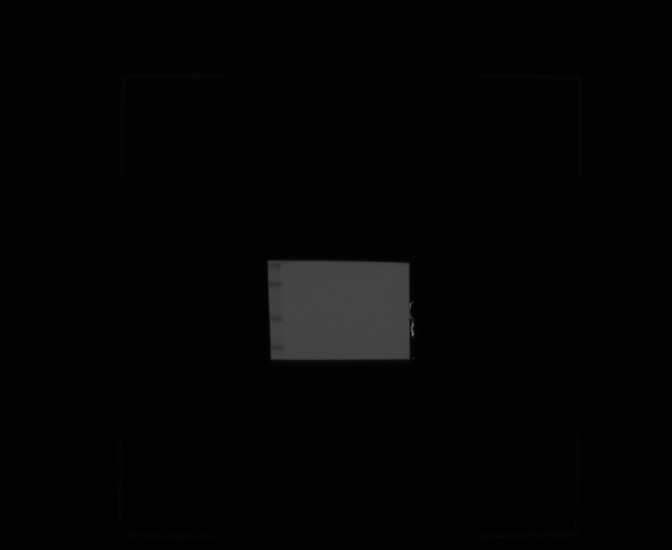

Supplement: Supplementary file 1 [file biomedicines-14-00403-s001.zip › a) WB original 1/AMPK-3_MARKER.tif]

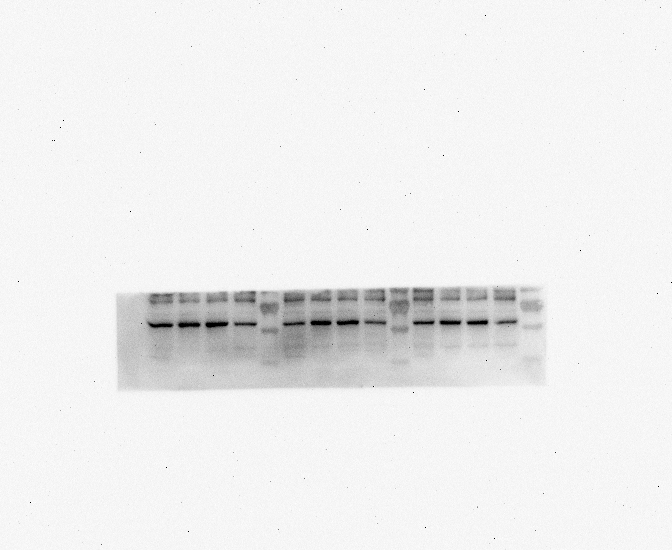

Supplement: Supplementary file 1 [file biomedicines-14-00403-s001.zip › a) WB original 1/PAMPK-1_.tif]

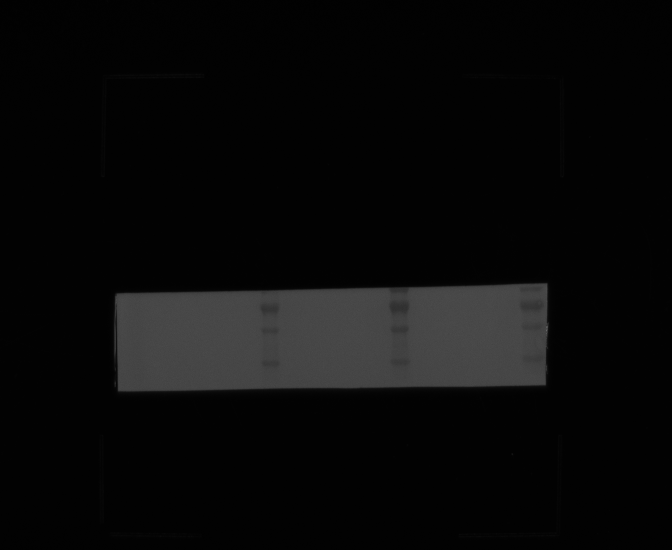

Supplement: Supplementary file 1 [file biomedicines-14-00403-s001.zip › a) WB original 1/PAMPK-1_MARKER.tif]

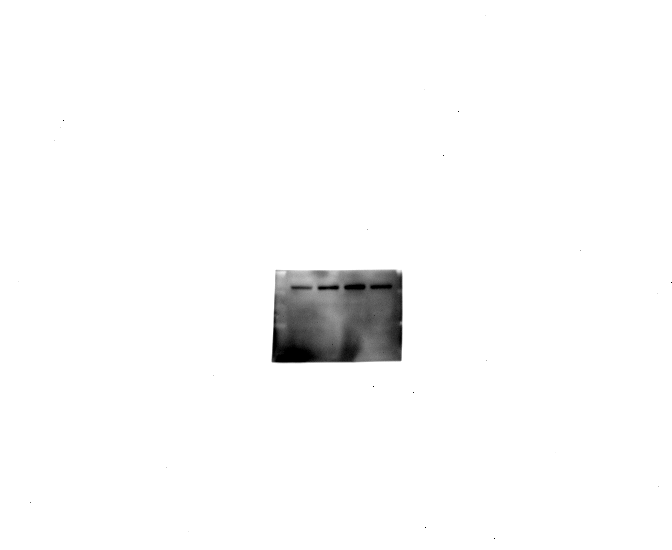

Supplement: Supplementary file 1 [file biomedicines-14-00403-s001.zip › a) WB original 1/pAMPK-2.tif]

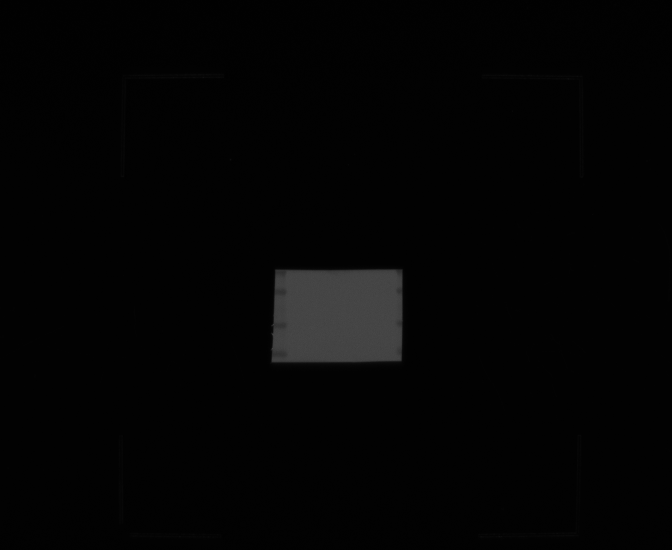

Supplement: Supplementary file 1 [file biomedicines-14-00403-s001.zip › a) WB original 1/pAMPK-2_MARKER.tif]

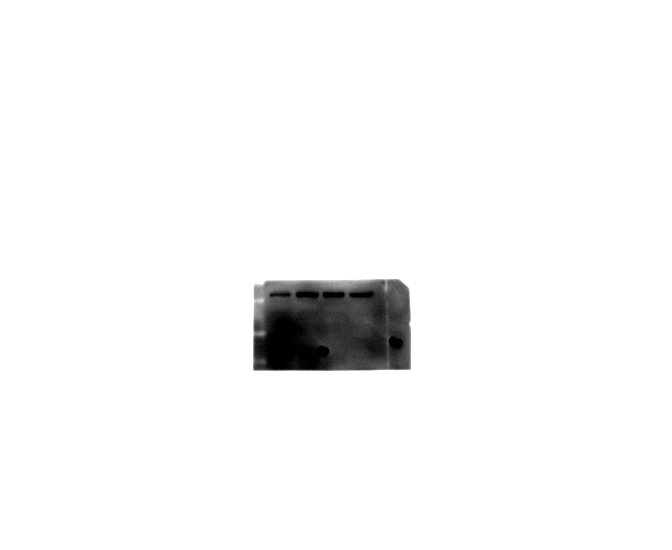

Supplement: Supplementary file 1 [file biomedicines-14-00403-s001.zip › a) WB original 1/pAMPK-3.tif]

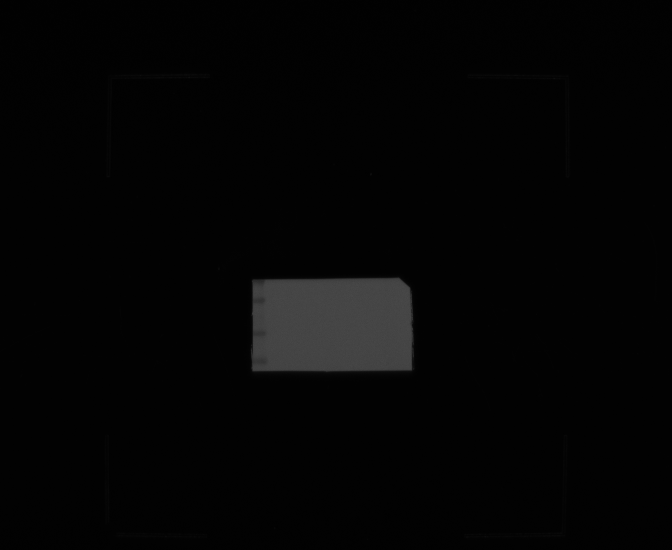

Supplement: Supplementary file 1 [file biomedicines-14-00403-s001.zip › a) WB original 1/pAMPK-3_MARKER.tif]

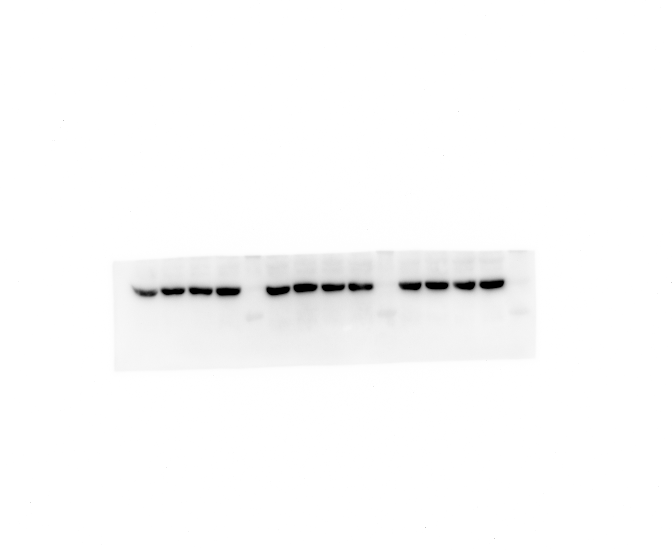

Supplement: Supplementary file 1 [file biomedicines-14-00403-s001.zip › a) WB original 1/β-actin-1_.tif]

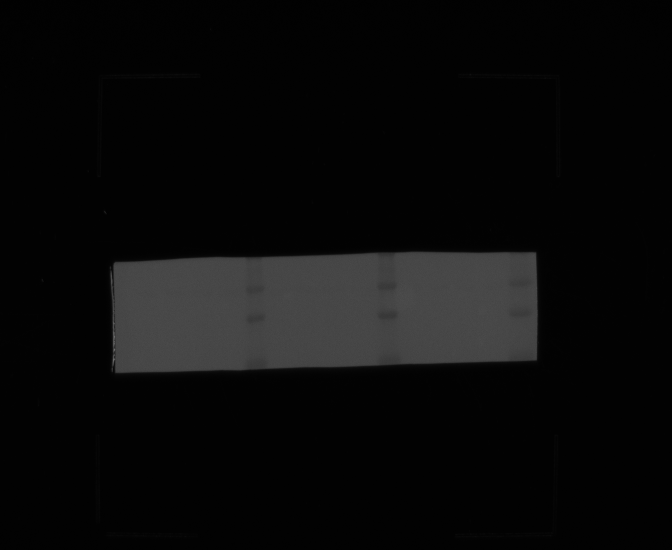

Supplement: Supplementary file 1 [file biomedicines-14-00403-s001.zip › a) WB original 1/β-actin-1_MARKER.tif]

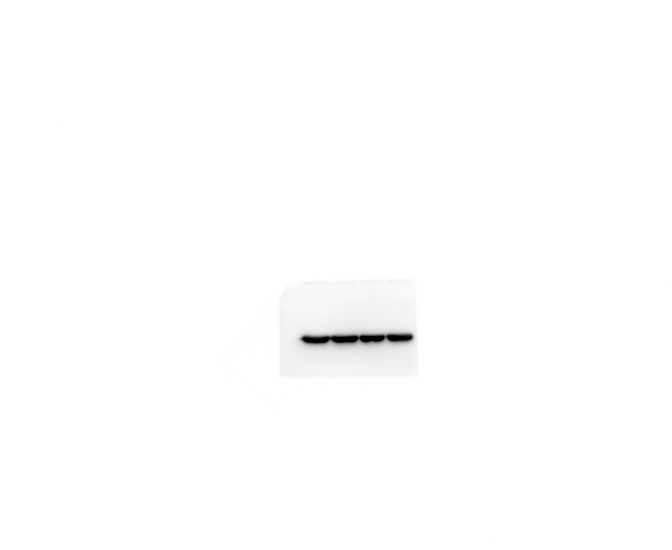

Supplement: Supplementary file 1 [file biomedicines-14-00403-s001.zip › a) WB original 1/β-actin-2.tif]

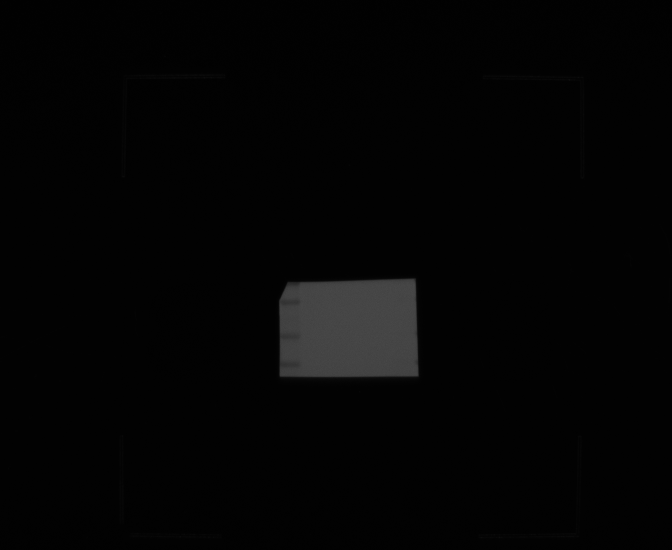

Supplement: Supplementary file 1 [file biomedicines-14-00403-s001.zip › a) WB original 1/β-actin-2_MARKER.tif]

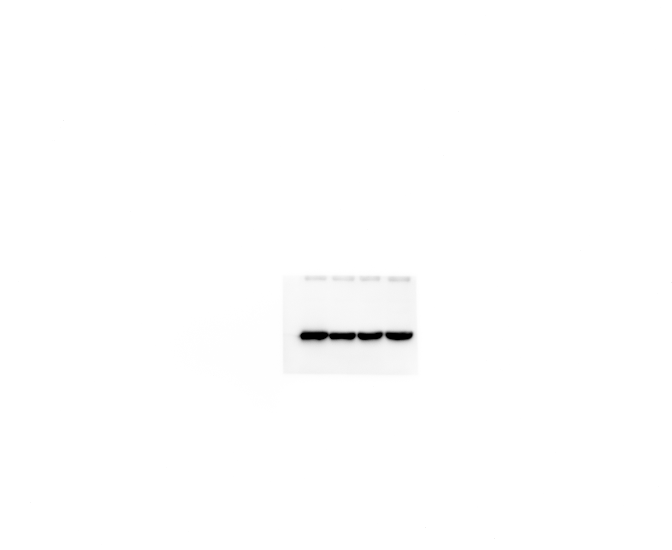

Supplement: Supplementary file 1 [file biomedicines-14-00403-s001.zip › a) WB original 1/β-actin-3.tif]

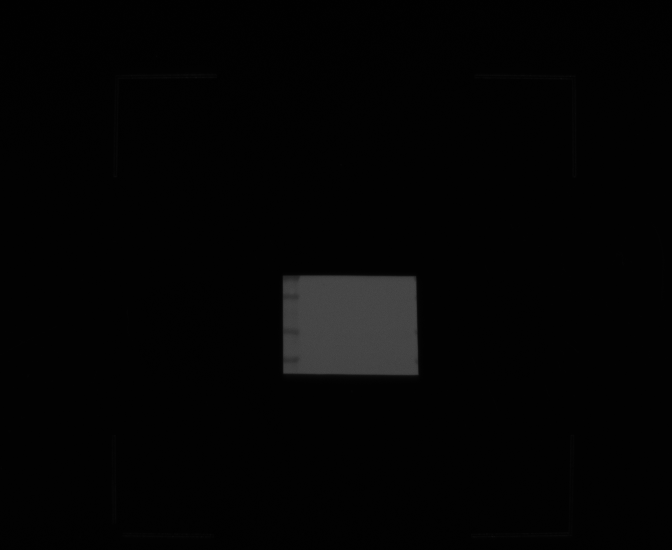

Supplement: Supplementary file 1 [file biomedicines-14-00403-s001.zip › a) WB original 1/β-actin-3_MARKER.tif]

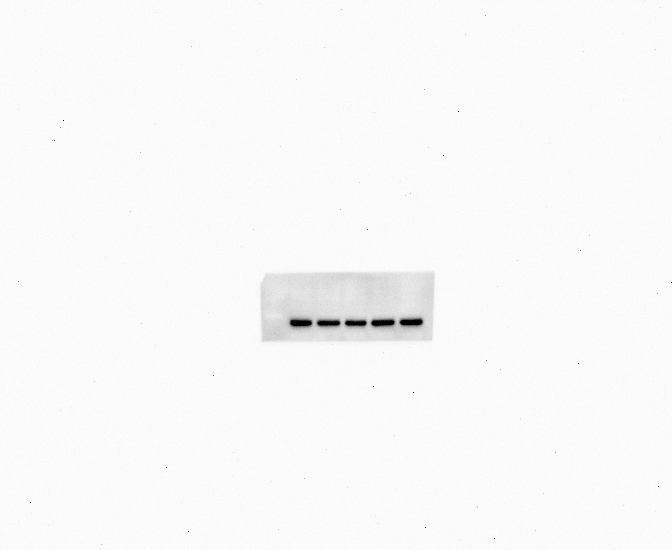

Supplement: Supplementary file 1 [file biomedicines-14-00403-s001.zip › b) WB original 2/AMPK1.tif]

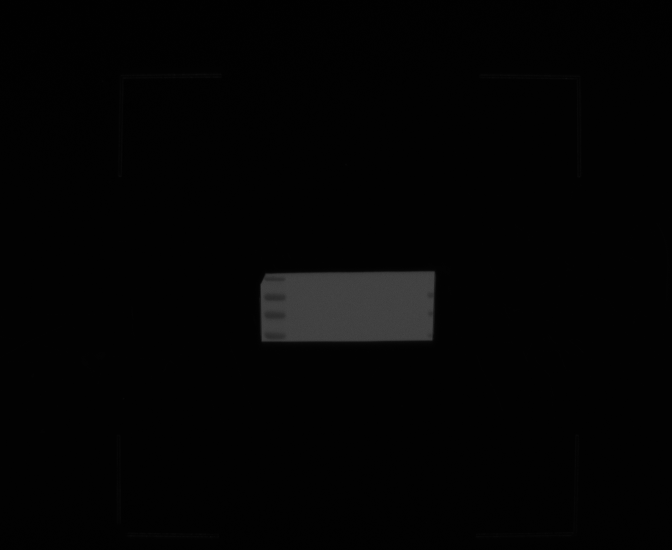

Supplement: Supplementary file 1 [file biomedicines-14-00403-s001.zip › b) WB original 2/AMPK1_MARKER.tif]

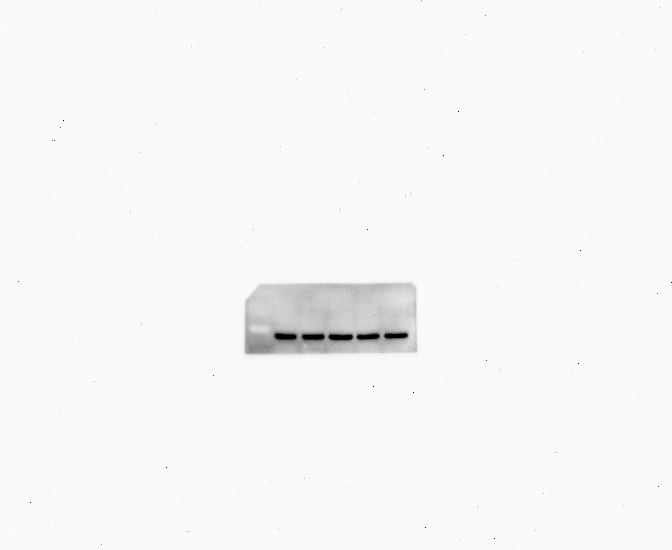

Supplement: Supplementary file 1 [file biomedicines-14-00403-s001.zip › b) WB original 2/AMPK2.tif]

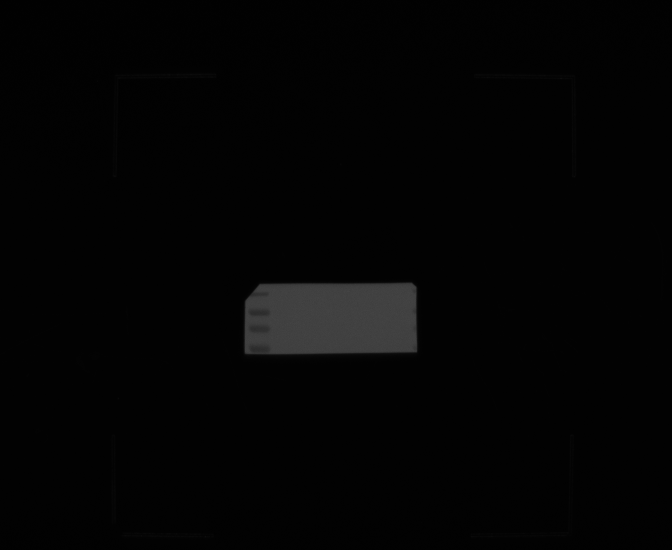

Supplement: Supplementary file 1 [file biomedicines-14-00403-s001.zip › b) WB original 2/AMPK2_MARKER.tif]

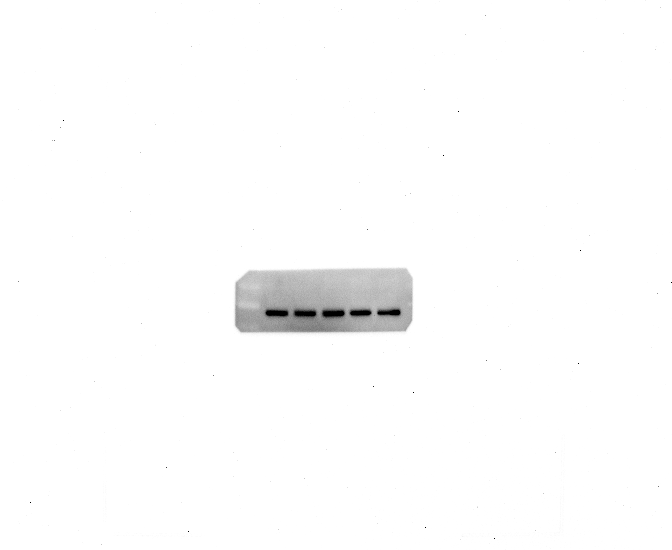

Supplement: Supplementary file 1 [file biomedicines-14-00403-s001.zip › b) WB original 2/AMPK3.tif]

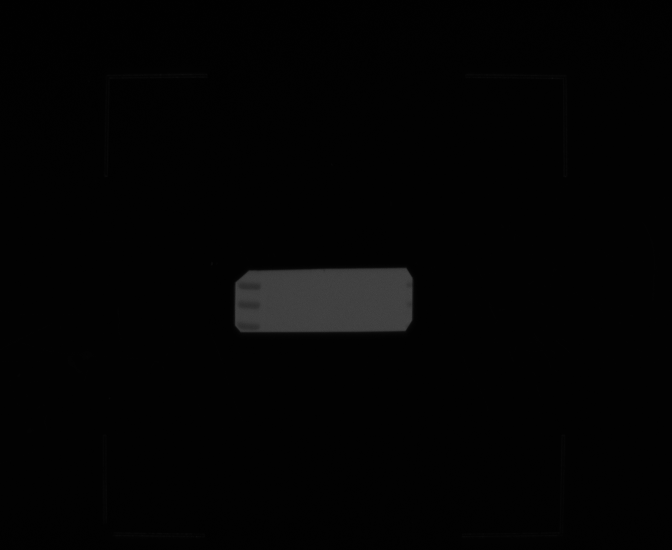

Supplement: Supplementary file 1 [file biomedicines-14-00403-s001.zip › b) WB original 2/AMPK3_MARKER.tif]

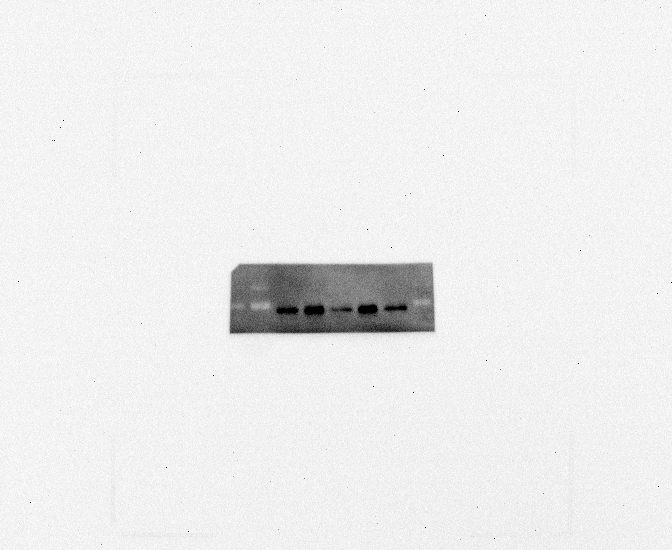

Supplement: Supplementary file 1 [file biomedicines-14-00403-s001.zip › b) WB original 2/PAMPK1.tif]

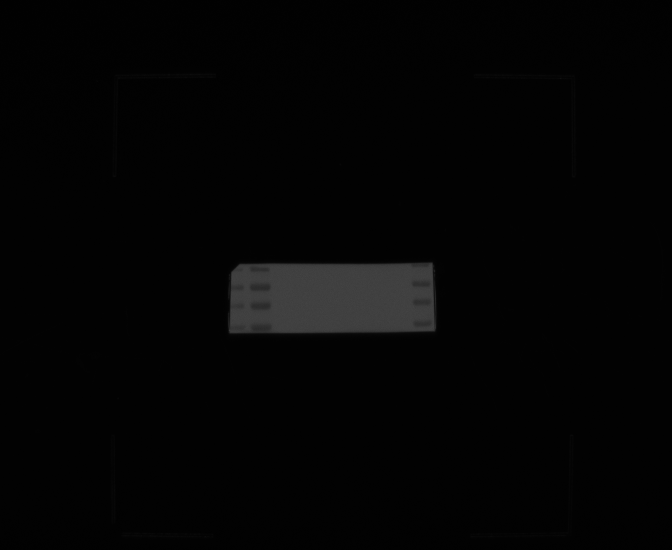

Supplement: Supplementary file 1 [file biomedicines-14-00403-s001.zip › b) WB original 2/PAMPK1_MARKER.tif]

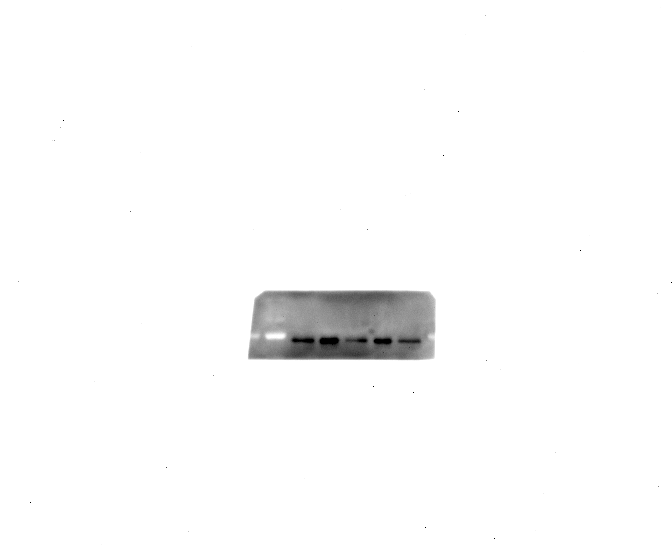

Supplement: Supplementary file 1 [file biomedicines-14-00403-s001.zip › b) WB original 2/PAMPK2.tif]

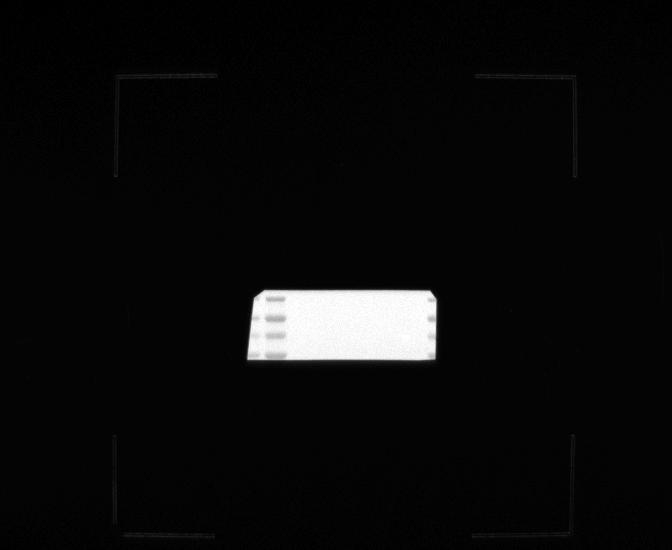

Supplement: Supplementary file 1 [file biomedicines-14-00403-s001.zip › b) WB original 2/PAMPK2_MARKER.tif]

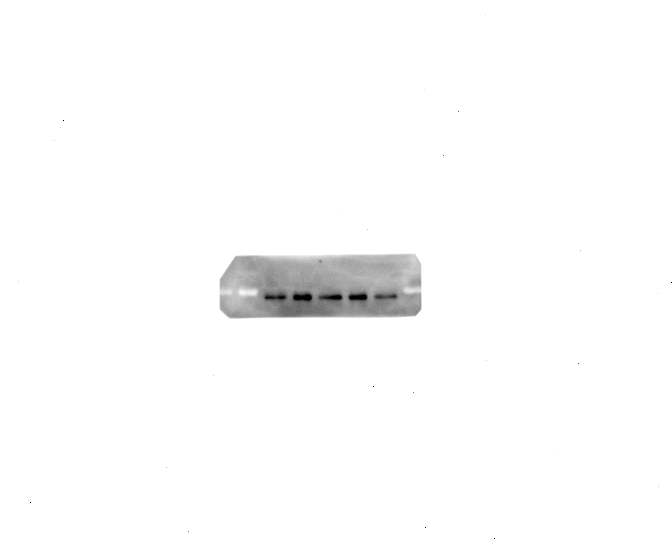

Supplement: Supplementary file 1 [file biomedicines-14-00403-s001.zip › b) WB original 2/PAMPK3.tif]

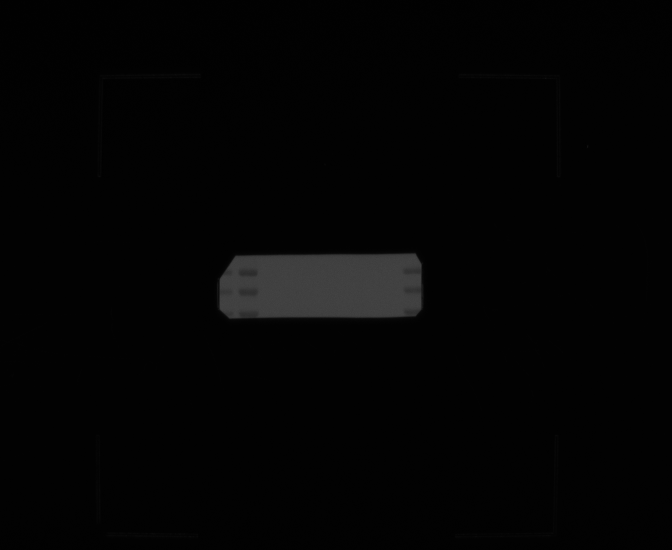

Supplement: Supplementary file 1 [file biomedicines-14-00403-s001.zip › b) WB original 2/PAMPK3_MARKER.tif]

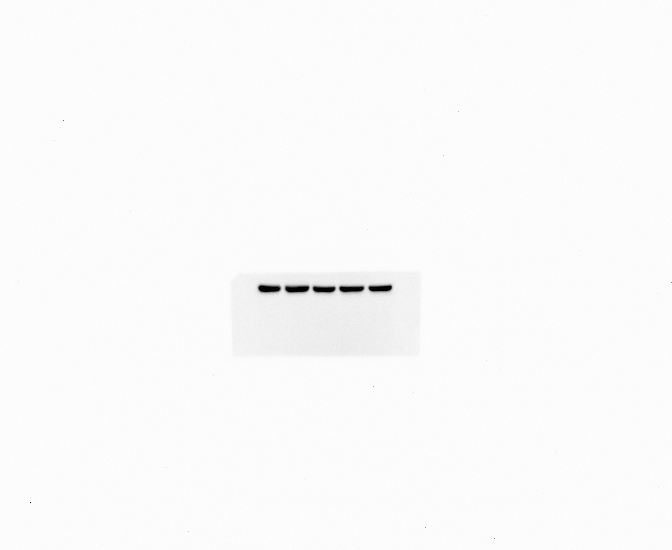

Supplement: Supplementary file 1 [file biomedicines-14-00403-s001.zip › b) WB original 2/β-ACTIN1.tif]

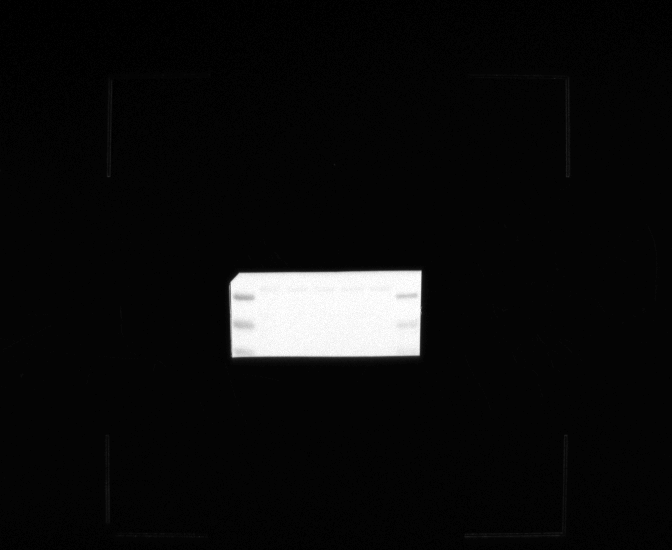

Supplement: Supplementary file 1 [file biomedicines-14-00403-s001.zip › b) WB original 2/β-ACTIN1_MARKER.tif]

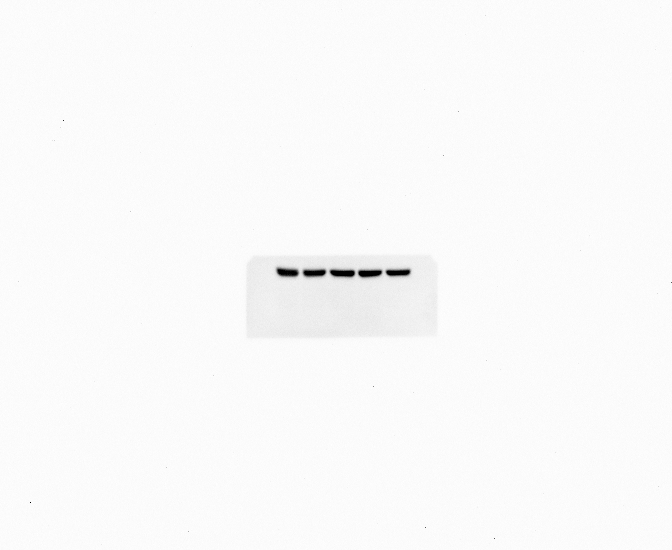

Supplement: Supplementary file 1 [file biomedicines-14-00403-s001.zip › b) WB original 2/β-ACTIN2.tif]

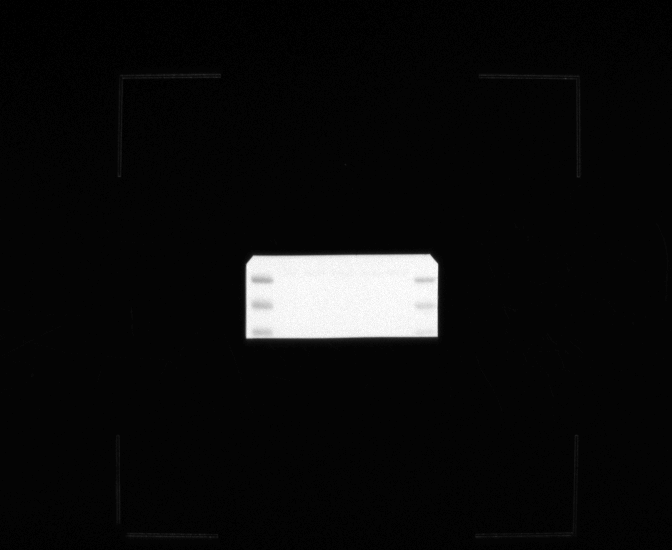

Supplement: Supplementary file 1 [file biomedicines-14-00403-s001.zip › b) WB original 2/β-ACTIN2_MARKER.tif]

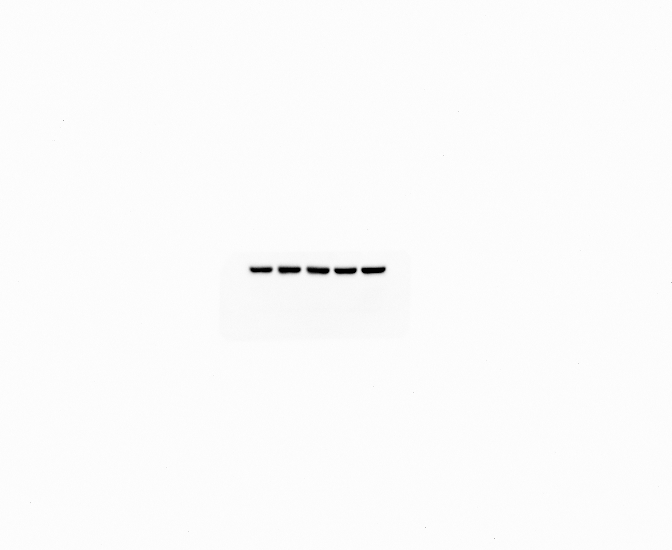

Supplement: Supplementary file 1 [file biomedicines-14-00403-s001.zip › b) WB original 2/β-ACTIN3.tif]

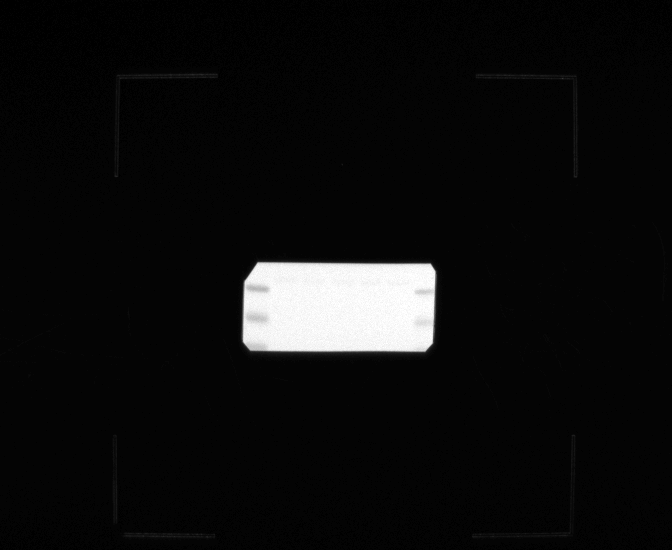

Supplement: Supplementary file 1 [file biomedicines-14-00403-s001.zip › b) WB original 2/β-ACTIN3_MARKER.tif]
